# Discovery of ferulic acid carbamate derivatives as dual-targeting agents of BuChE and Nrf2 for Alzheimer’s disease

**DOI:** 10.1080/14756366.2026.2645483

**Published:** 2026-03-23

**Authors:** Kejing Lao, Yingze Li, Yueyan Xiao, Ya Sun, Yuxuan Dai, Huijin Li, Yang Yang, Yun Zhang, Jing Wang, Weize Li, Xingchun Gou, Li Guan

**Affiliations:** ^a^ Shaanxi Key Laboratory of Brain Disorders, Institute of Basic and Translational Medicine, Xi’an Medical University, Xi’an, China; ^b^ College of Pharmacy, Xi’an Medical University, Xi’an, China

**Keywords:** Alzheimer’s disease, BuChE inhibitor, ferulic acid, Nrf2 pathway

## Abstract

Given the multifactorial aetiology of Alzheimer’s disease, multi-target strategies have emerged as a promising therapeutic approach. In this study, we designed and synthesised a series of ferulic acid carbamate derivatives to selectively inhibit BuChE and stimulate Nrf2 pathway. The biological evaluation revealed that compound **5c** and **5e** were the most potent, exhibiting over 150-fold selectivity for BuChE. Also, **5c**, **5g** and **5h** significantly reversed both H_2_O_2_^−^ and Aβ-induced toxicity in HT22 cells. These compounds were further shown to eliminate ROS accumulation induced by Aβ and upregulated HO-1 and GCLM by promoting the nuclei translocation of Nrf2. In Aβ transgenic *C. elegans*, three lead compounds alleviated Aβ-induced paralysis and cognitive deficits. *In silico* study revealed that compound **5c** fitted well into the active sites of BuChE and Keap1 while maintaining favourable CNS drugability. This dual strategy of cholinesterase inhibition and oxidative stress mitigation is a promising approach for novel AD therapeutics.

## Graphical Abstract

**Figure:**
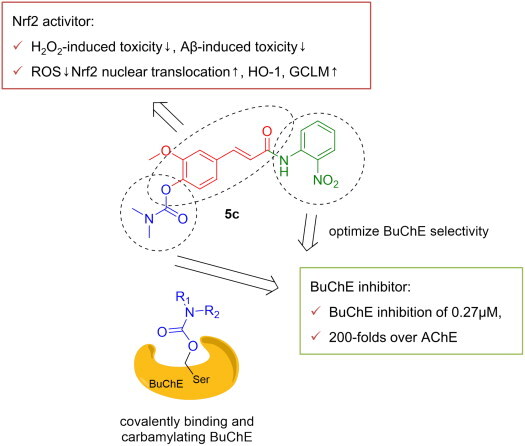

## Introduction

Alzheimer’s disease (AD), a progressive neurodegenerative disorder, represents the predominant cause of memory impairment and dementia in the elderly population. It was indicate that, and this number may escalate to 13.8 million by 2060 in the absence of medical breakthroughs^1^. AD imposes substantial socioeconomic burdens through direct healthcare costs and indirect caregiver impacts, compounded by profound psychological distress for affected families.

AD is neuropathologically characterised by the loss of neurons and synapses in the cerebral cortex and certain subcortical regions. While the precise pathogenesis remains controversial, current understanding implicates multiple interacting mechanisms including amyloid-β (Aβ) aggregation, tau hyperphosphorylation, neuroinflammatory processes, and neuronal death^2^. The inherent complexity and multifactorial aetiology of AD has contributed to high failure in drug development, with numerous single-target agents failing clinical trials due to insufficient efficacy or adverse effects. Given the complicated multifactorial network between molecular targets across diverse pathological pathways, have emerged as pivotal and effective approaches which consequently gained increasing interest in Alzheimer’s disease drug development^3^.

Within the framework of the cholinergic hypothesis for Alzheimer’s disease (AD), increasing synaptic acetylcholine (ACh) levels is a key strategy for improving cognitive function^4^. In humans, the hydrolysis of ACh is primarily carried out by two enzymes: acetylcholinesterase (AChE) and butyrylcholinesterase (BuChE). Although AChE inhibitors have been used clinically for many years, their efficacy in moderate to severe stages of AD remains limited^5^. Notably, during disease progression, cortical AChE activity decreases significantly, whereas BuChE activity rises markedly, with the BuChE/AChE ratio increasing from approximately 0.5 to 11 in the late stages^6^. This shift indicates that BuChE becomes the predominant cholinesterase in advanced AD, making it a compelling therapeutic target. Selective inhibition of BuChE offers potential advantages over conventional AChE inhibitors: the latter are often associated with peripheral side effects such as gastrointestinal disturbances and reduced appetite, which are less frequently observed with BuChE-selective inhibitors^7^. Furthermore, BuChE is involved in AD pathological processes including Aβ deposition and neuroinflammation, suggesting that its inhibition may exert disease-modifying effects. Although BuChE also plays physiological roles in cocaine addiction^8^, detoxification^9^, and lipid regulation^10^, which raising concerns about potential off-target effects with prolonged inhibition, its pronounced upregulation in late-stage AD and its involvement in disease pathology support the rationale for developing highly selective BuChE inhibitors as a novel treatment strategy for moderate to severe AD. Future research should further balance efficacy and safety to optimise drug design for precise intervention.

Emerging evidence positions oxidative stress as both a contributor to and consequence of AD progression, establishing a self-perpetuating cycle through bidirectional interactions with Aβ pathology. Aβ aggregates stimulate reactive oxygen species (ROS) overproduction, initiating cascades of DNA damage, apoptotic signalling, and neurotoxic neuroinflammation via microglial activation^11^. Notably, oxidative stress reciprocally enhances Aβ deposition and tau hyperphosphorylation, creating a pathogenic feedback loop that exacerbates neurodegeneration^12^.

The Keap1-Nrf2-ARE signalling axis serves as a main regulator of cellular redox homeostasis, orchestrating the expression of cytoprotective enzymes including NAD(P)H:quinone reductase (NQO1), haem oxygenase-1 (HO-1), and glutathione S-transferase (GST)^13^. Despite extensive oxidative damage in AD brains, a diminished expression of Nrf2-mediated antioxidant defences has been demonstrated, suggesting therapeutic potential in pharmacological Nrf2 activation^14^.

Ferulic acid (FA), a ubiquitous 4-hydroxycinnamic acid derivative abundant in plant-based foods, demonstrates multimodal neuroprotective properties encompassing antioxidant, anti-inflammatory, and anti-amyloid activities^15^. Mechanistic studies attribute its bioactivity to the α,β-unsaturated carbonyl moiety that covalently modifies Keap1, thereby stabilising Nrf2 and enhancing antioxidant response element (ARE)-driven gene transcription^16^. However, clinical translation of FA has been limited by pharmacokinetic limitations including poor blood-brain barrier permeability, rapid metabolism, and chemical instability.

Extensive efforts have been devoted to overcoming the inherent limitations of FA—such as its weak cholinesterase inhibitory activity, poor brain permeability, and low water solubility—through structural modification, yielding a plethora of FA-based derivatives investigated as MTDLs for AD^17–19^. These synthetic strategies can be broadly categorised into several approaches. One predominant approach involves the hybridisation of the FA scaffold with pharmacophores from approved anti-AD drugs, such as donepezil^20^^,^^21^, tacrine^22^, rivastigmine^23^^,^^24^, and memantine^25^, aiming to combine FA’s antioxidant/anti-Aβ properties with enhanced cholinergic inhibition. Another strategy focuses on conjugating FA with other privileged structural motifs known for specific bioactivities, including 1,2,3,4-tetrahydroisoquinoline for BuChE/MAO inhibition^26^ and carbazole for ChE inhibition^27^. Furthermore, direct structural modifications on the FA core have been explored to improve drug-like properties, such as synthesising water-soluble glycerol esters to enhance solubility^28^, PEGylating to improve BBB penetration^29^, or converting the acid group to amides and esters to modulate lipophilicity^30^. While many of these derivatives exhibit improved potency in inhibiting particularly AChE, modulating Aβ aggregation, or mitigating oxidative stress *in vitro*, the strategic design of a molecule that intentionally and efficiently couples a selective BuChE inhibitory mechanism with the potent activation of endogenous cytoprotective pathways, such as Keap1-Nrf2, remains a significant innovation frontier.

In this study, we designed a novel series of FA derivatives based on a dual-target synergistic strategy that fundamentally departs from conventional multi-target inhibitor design. Rather than simply hybridising pharmacophores, we sought to intrinsically embed two complementary mechanisms within a single, cohesively modified FA scaffold: selective BuChE inhibition for cholinergic symptom relief and potent activation of the Keap1-Nrf2 antioxidant pathway for endogenous neuroprotection. This approach aims to create a mechanism-synergistic agent rather than a mere combination of inhibitory activities. To robustly validate the therapeutic potential of this strategy, we employed transgenic C. elegans AD models (CL4176 and CL2355) for in vivo functional assessment. This represents a significant advancement, as it moves beyond *in vitro* assays to demonstrate direct rescue of Aβ-induced paralysis and cognitive-behavioural deficits in a whole organism—a level of functional validation rarely applied to FA-based BuChE inhibitors and crucial for establishing disease-modifying potential.

## Results and discussion

### Design and synthesis

**Figure 1.**
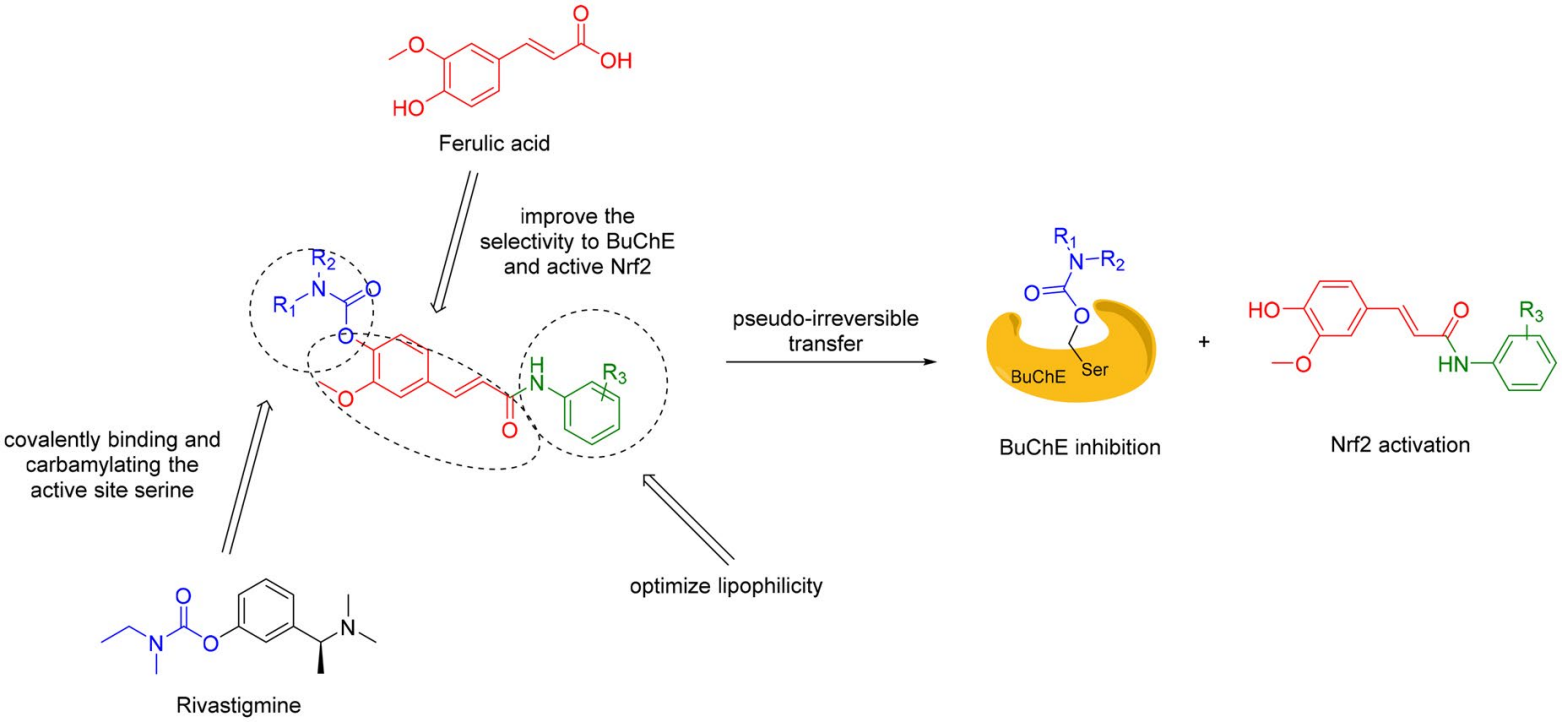
The design of FA derivatives and its dual pseudo-irreversible BuChE inhibition and Nrf2 activation activity.

The design of target compounds embodies a rational and precise functionalization of the FA core to achieve our dual-target strategy. Rivastigmine, an effective and selective BuChE inhibitor highly successed in both clinical and pharmacological investigations, is a pseudo-irreversible inhibitor by covalently binding and carbamylating the active site serine^31^. Considering the differences of the acyl-binding pockets of AChE and BuChE crystal structures, it was identified that BuChE could tolerate larger substrates because it was provided a wider space in acyl-binding pocket^32^. Thus, to optimise physicochemical properties and selectivity towards the BuChE active site, the carboxylic acid was conjugated with various aniline derivatives, which enabled the modulation of lipophilicity and binding without the introduction of large, rigid auxiliary fragments commonly employed in prior designs. Throughout these modifications, the α,β-unsaturated carbonyl system of FA was preserved to maintain the potential for Keap1 interaction and Nrf2 activation. Consequently, FA is transformed from a simple building block into an integrated multifunctional core, wherein BuChE inhibition is enabled by the carbamate moiety, structural optimisation is facilitated by the anilide group, and Nrf2 activation is driven by the intact cinnamoyl backbone. This represents a strategic shift from pharmacophore hybridisation towards the integration of inherent mechanisms (Figure 1).

**Scheme 1.**
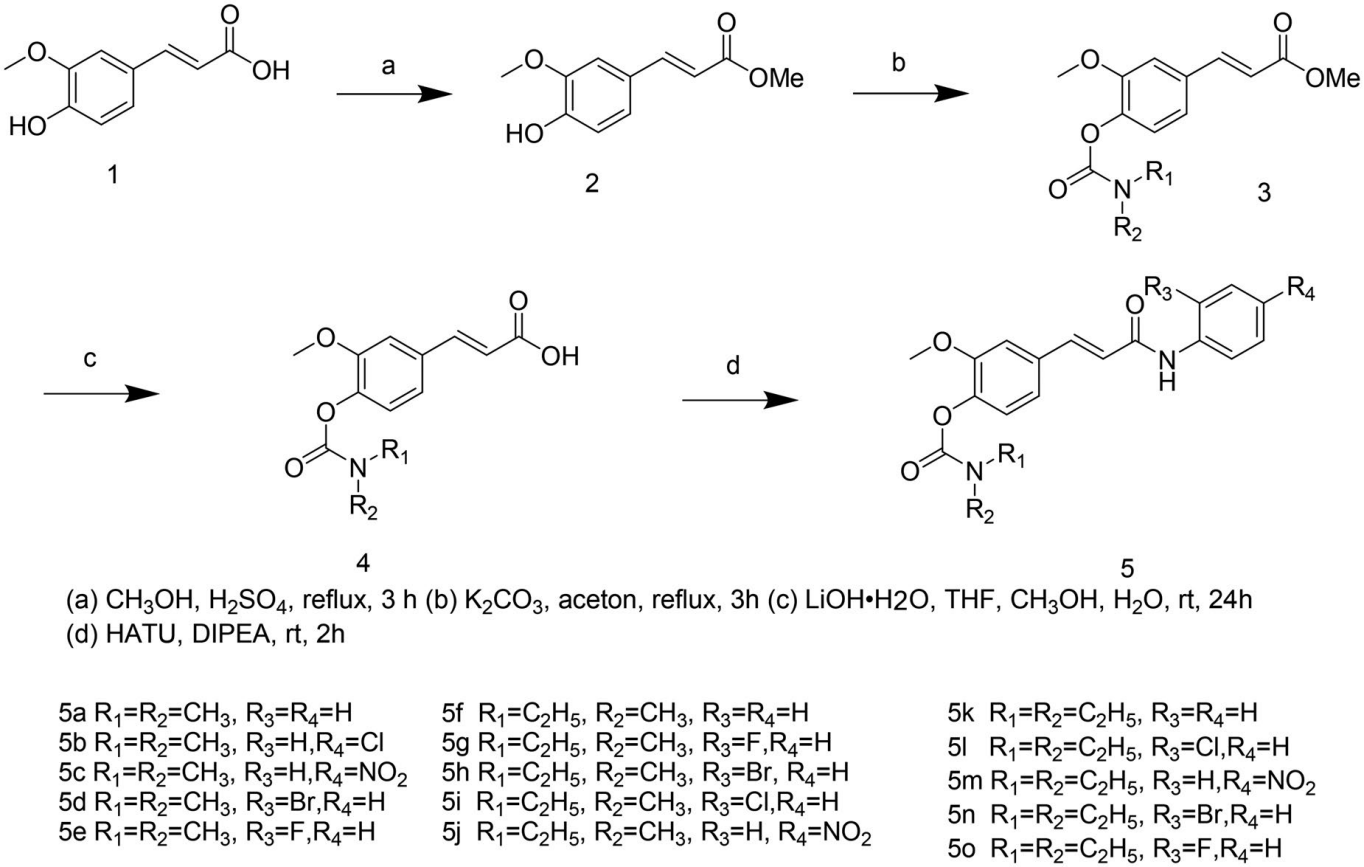
Synthetic route of compounds 5a**–**5o.

The synthetic routes for the designed compounds **5a–5o** were shown in Scheme 1. By taking FA as the initial substrate, the carboxylic acid group was protected as methyl ester in methanol. Subsequently, the corresponding carbamoyl chloride derivatives were selectively coupled with the phenolic hydroxyl in the presence of K_2_CO_3_ as base. The methyl ester protecting group was then selectively hydrolysed under mild alkaline conditions using lithium hydroxide in a THF/H_2_O mixture at r.t. The activated carboxylic acid intermediate was condensed with the corresponding aniline derivatives by HATU/DIPEA in DCM to afford the target compound.

### Cholinesterase inhibition activity

The inhibition activity of AChE and BuChE were fist evaluated by taking rivastigmine as positive control. As shown in Table 1, most compounds displayed better inhibitory activity to BuChE than AchE a trend likely attributable to the structural differences in their active site gorges, with BuChE possessing a deeper and more hydrophobic cavity that better accommodates the scaffold of this series.

**Table 1.** The AChE and BuChE inhibition activity of compounds **5a–5o**.

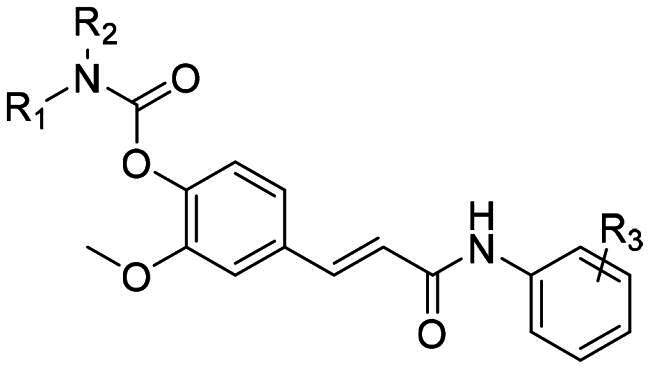							
Compounds	R_1_	Ring A	AChE Inhibition		BuChE Inhibition		SI^b^
			50 μM (I%)	IC_50_ (μM)	50 μM (I%)	IC_50_ (μM)	
5d	R_1_,R_2_ = CH_3_	R_3_ = 2-Br	13.92	-^a^	88.36	0.62	80.64
**Rivastigmine**	–	–	88.92	2.52	73.68	0.13	19.38
**5a**	R_1_,R_2_ = CH_3_	R_3_ = H	21.91	-^a^	53.56	8.21	6.09
**5b**	R_1_,R_2_ = CH_3_	R_3_ = 4-NO_2_	24.47	-^a^	72.91	1.79	27.93
**5c**	R_1_,R_2_ = CH_3_	R_3_ = 2-F	46.35	-^a^	93.80	0.27	185.18
**5e**	R_1_,R_2_ = CH_3_	R_3_ = 4-Cl	21.43	-^a^	90.55	0.17	294.10
**5f**	R_1_ = CH_3_, R_2_ = C_2_H_5_	R3 = H	18.98	-^a^	88.00	0.32	156.25
**5g**	R_1_ = CH_3_, R_2_ = C_2_H_5_	R_3_ = 4-NO_2_	30.80	-^a^	72.24	5.06	9.88
**5h**	R_1_ = CH_3_, R_2_ = C_2_H_5_	R_3_ = 2-Cl	13.60	-^a^	61.16	8.34	5.99
**5i**	R_1_ = CH_3_, R_2_ = C_2_H_5_	R_3_ = 2-F	24.00	-^a^	67.32	2.38	21.00
**5j**	R_1_ = CH_3_, R_2_ = C_2_H_5_	R_3_ = 2-Br	46.20	-^a^	63.08	6.58	7.59
**5k**	R_1_,R_2_ = C_2_H_5_	R3 = H	28.88	-^a^	22.78	-^a^	-^c^
**5l**	R_1_,R_2_ = C_2_H_5_	R_3_ = 4-NO_2_	22.45	-^a^	19.32	-^a^	-^c^
**5m**	R_1_,R_2_ = C_2_H_5_	R_3_ = 2-Cl	34.34	-^a^	12.32	-^a^	-^c^
**5n**	R_1_,R_2_ = C_2_H_5_	R_3_ = 2-F	13.93	-^a^	22.41	-^a^	-^c^
**5o**	R_1_,R_2_ = C_2_H_5_	R_3_ = 2-Br	13.57	-^a^	29.63	-^a^	-^c^

^a^ IC_50_ >50 μM.

^b^ SI (selective index) = IC_50(AChE)_/IC_50(BuChE)_, For those IC_50_ of AchE over 50 μM, the IC_50(AChE)_ was calculated as 50 μM.

^c^ SI cannot been calculated.

**Figure 2.**
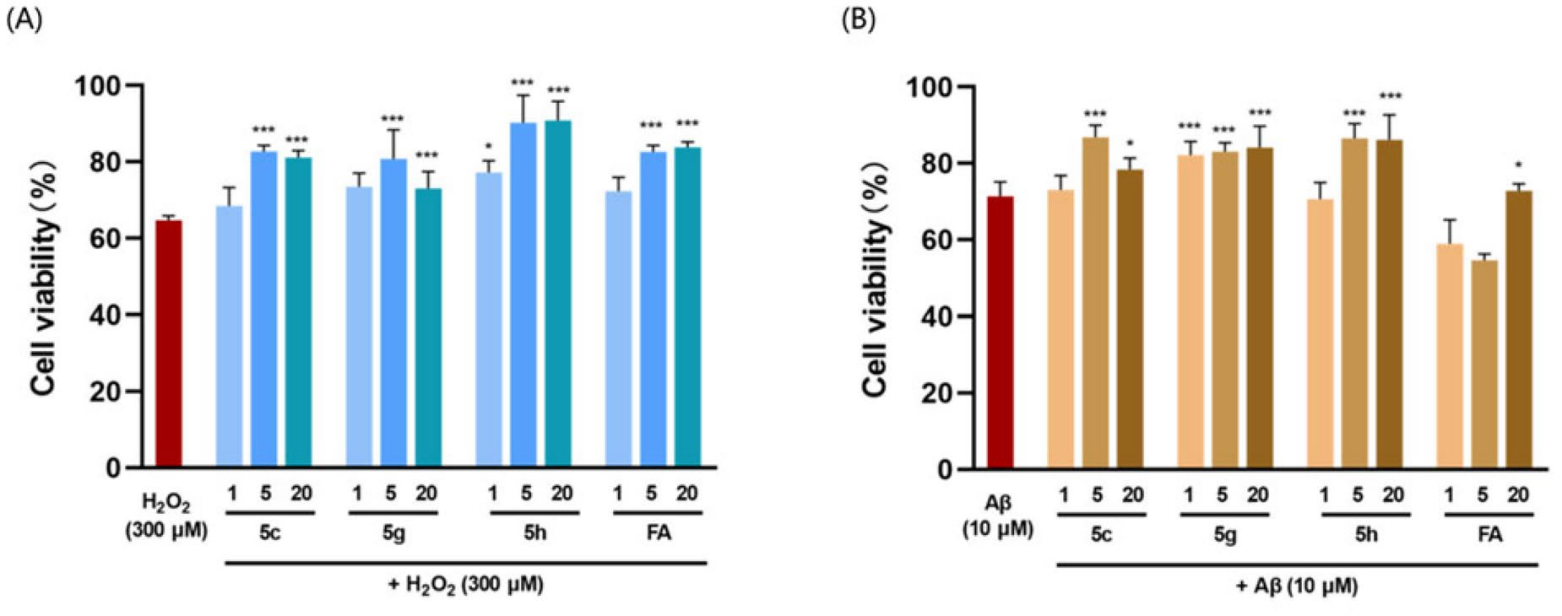
The neuroprotective effect of compounds **5c**, **5g**, **5h** and **FA** in H_2_O_2_-induced model (A) and Aβ-induced model (B) in HT22 cells. HT22 cells were cultured with different concentrations of tested compounds with H_2_O_2_ (300 μM) and Aβ (10 μM) separately. Values are mean ± SD (*n* = 3). *p < 0.05 vs. model group, ***p < 0.001 vs. model group.

The amino substituent (R^1^, R^2^) critically influences BuChE inhibition. Compounds featuring a dimethylamino group consistently exhibited potent BuChE inhibition, with IC_50_ values in the low micromolar to sub-micromolar range. Notably, compounds 5c (IC_50_ = 0.27 μM) and 5e (IC_50_ = 0.17 μM) displayed activity comparable to the positive control rivastigmine (IC_50_ = 0.13 μM), suggesting optimal steric complementarity and potential cation-π interactions within the BuChE active site. In contrast, enlargement of this group to a diethylamino moiety generally led to a marked decrease in potency, as observed for compounds 5 g**–**5j (IC_50_ = 2.38**–**8.34 μM). Furthermore, derivatives with two ethyl substituents (5k**–**5o) lost significant activity (IC_50_ > 50 μM), underscoring that excessive steric bulk around the amino nitrogen is detrimental, likely by hindering optimal penetration into the gorge or disrupting orientation towards key catalytic residues (Figure 2).

Modifications on the phenyl ring (Ring A, R³) provided fine-tuning of activity. The introduction of electron-withdrawing groups generally enhanced BuChE inhibition. Halogen substitutions at the 2- or 4-positions, as in 2-fluoro and 4-chloro derivatives (5c and 5e), yielded the most potent compounds. This enhancement is potentially mediated by increased electron deficiency of the aromatic ring, strengthening π-π stacking or anion-π interactions with aromatic residues in the active site, while the small size of fluorine favours deep pocket penetration. The 4-nitro group (5b and 5 g) also conferred good activity, likely through a similar electronic effect, though its slightly lower potency compared to halogens may indicate suboptimal fit due to its larger size or polarity. Unsubstituted or weaker analogues suggest that a higher electron density on the aromatic ring offers less favourable electrostatic complementarity.

Thus, dimethylamino is essential for high BuChE potency, while electron-withdrawing substituents, particularly F or Cl, at specific positions on the pendant aryl ring significantly enhance both activity and selectivity, presumably by optimising interactions within the BuChE gorge. Notably, compound 5c and 5e emerges as the most balanced candidate most balanced candidate, exhibiting both potent inhibition (IC_50_ of 0.27 and 0.17 μM respectively) and high selectivity (SI of 185.18 and 294.14 respectively)

### In vitro neuroprotective effect on HT22 cell

To evaluate the neuroprotective potential of the synthesised compounds, their safety was first assessed.As shown in Table 2, the majority of compounds exhibited very low cytotoxicity at 50 μM, with cell viability exceeding 90% in most cases. Only compounds **5b**, **5i** and **5 m** showed slightly reduced viability.

**Table 2.** The safety and neuroprotective effect of compounds **5a–5o**.

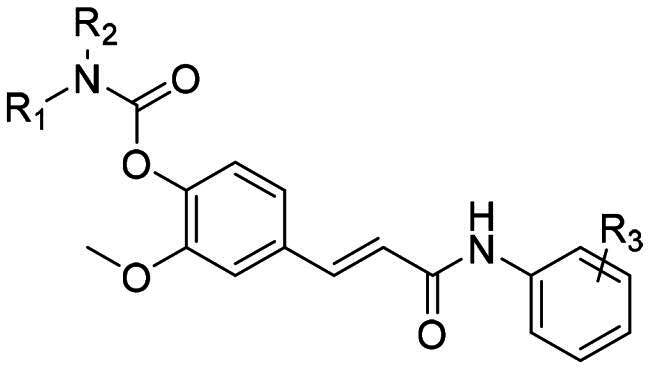					
Compounds	R_1_	Ring A	Safety (50 μM,%)	Neuroprotective effect (20 μM, %)	
				H_2_O_2_-induced inhibition	Aβ-induced inhibition
Model	–	–	–	64.02 ± 2.12	71.36 ± 1.58
**5a**	R_1_,R_2_ = CH_3_	R_3_ = H	98.48 ± 2.25	75.79 ± 6.48	54.81 ± 4.17
**5b**	R_1_,R_2_ = CH_3_	R_3_ = 4-NO_2_	72.25 ± 5.44	70.66 ± 5.25	47.64 ± 7.41
**5c**	R_1_,R_2_ = CH_3_	R_3_ = 2-F	96.39 ± 2.30	81.10 ± 5.43	78.35 ± 2.77
**5d**	R_1_,R_2_ = CH_3_	R_3_ = 2-Br	89.40 ± 2.79	64.80 ± 8.18	67.94 ± 1.96
**5e**	R_1_,R_2_ = CH_3_	R_3_ = 4-Cl	95.26 ± 4.09	53.57 ± 2.77	69.95 ± 4.21
**5f**	R_1_ = CH_3_, R_2_ = C_2_H_5_	R_3_ = H	96.39 ± 10.00	63.08 ± 4.02	66.21 ± 1.73
**5g**	R_1_ = CH_3_, R_2_ = C_2_H_5_	R_3_ = 4-NO_2_	93.46 ± 7.77	86.52 ± 6.62	84.15 ± 1.45
**5h**	R_1_ = CH_3_, R_2_ = C_2_H_5_	R_3_ = 2-Cl	98.40 ± 6.36	84.54 ± 1.82	86.12 ± 5.32
**5i**	R_1_ = CH_3_, R_2_ = C_2_H_5_	R_3_ = 2-F	77.65 ± 9.18	76.19 ± 2.48	67.02 ± 10.59
**5j**	R_1_ = CH_3_, R_2_ = C_2_H_5_	R_3_ = 2-Br	95.05 ± 5.47	74.20 ± 4.48	36.32 ± 3.84
**5k**	R_1_,R_2_ = C_2_H_5_	R3 = H	91.32 ± 7.06	44.1 ± 9.82	21.25 ± 8.82
**5l**	R_1_,R_2_ = C_2_H_5_	R_3_ = 4-NO_2_	92.06 ± 5.47	54.66 ± 2.81	44.82 ± 6.68
**5m**	R_1_,R_2_ = C_2_H_5_	R_3_ = 2-Cl	77.12 ± 4.69	41.40 ± 1.25	46.83 ± 3.36
**5n**	R_1_,R_2_ = C_2_H_5_	R_3_ = 2-F	98.52 ± 4.23	49.67 ± 11.37	35.42 ± 5.37
**5o**	R_1_,R_2_ = C_2_H_5_	R_3_ = 2-Br	95.66 ± 2.75	53.56 ± 12.79	54.02 ± 4.31
**FA**	–	–	93.59 ± 1.30	87.09 ± 1.22	72.83 ± 4.95

H_2_O_2_-induced oxidative damage is one of the most representative methods for the preliminary screening of compounds capable of scavenging activated oxygen species. All the synthesised compounds were tested for their cell protective effects in HT22 cell lines against H_2_O_2_ (Table 2). By treating with 300 μM H_2_O_2_, the cell viability was 64.02% of that in control group. The neuroprotective effect against H_2_O_2_ was evaluated by treating with both synthesised compounds (20 μM) and H_2_O_2_ (300 μM). As the positive control, FA elevated cell viability to 78.50%, consistently with previously reported data. Among the synthesised compounds, the best effects were observed on compounds **5c**, **5 g** and **5h** with the cell viability more than 85%.

**Figure 3.**
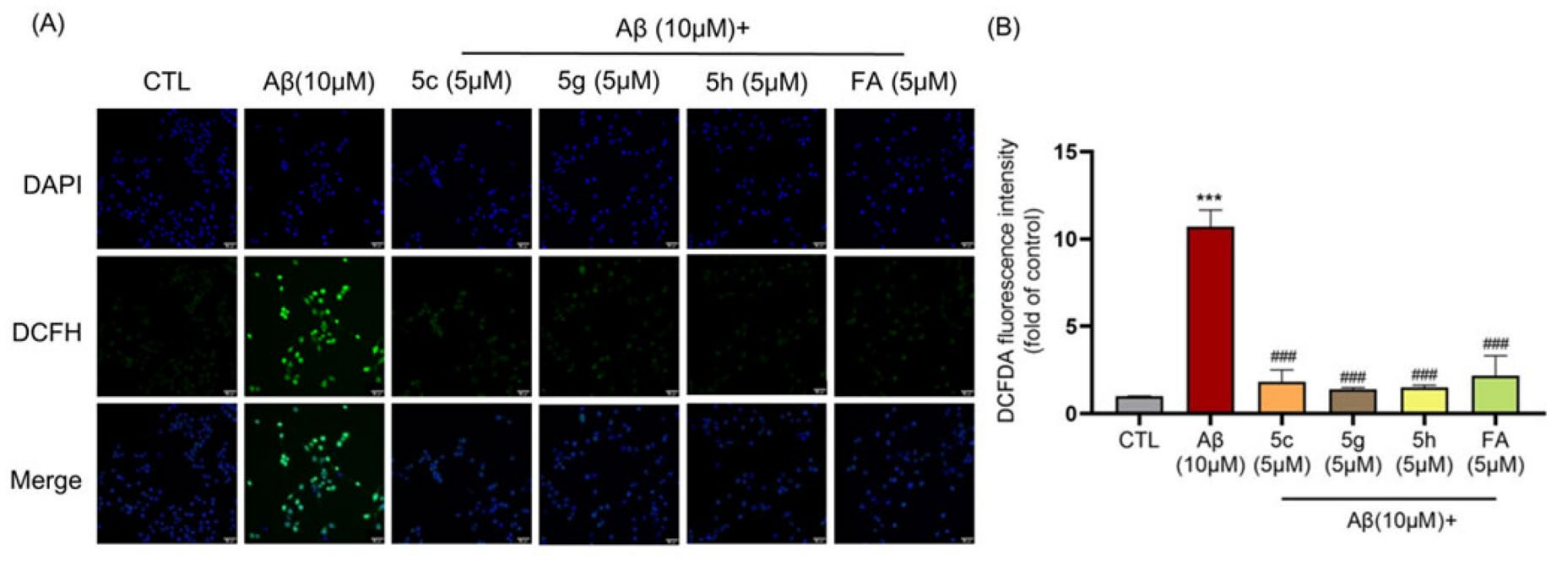
Compound **5c, 5g, 5h** and **FA** could eliminate the ROS accumulation induced by Aβ. (A) Representative images of HT22 cells stained by DCFH-DA. Scale bar, 50 µm. (B) Quantification of intracellular ROS levels. ***p < 0.005 vs the control group; ### *p* < 0.001 vs the Aβ group.

Since Aβ been recognised as the initiating of neurodegenerative cascade, the neuroprotective effect against Aβ of all the synthesised compounds also been evaluated. As shown in Table 2, by treating with 10 μM Aβ, the cell viability was 71.36% of that in control group. The neuroprotective effect against Aβ was evaluated by treating with both synthesised compounds (20 μM) and Aβ (10 μM). As the positive control, FA elevated cell viability to 72.83%. Among the synthesised compounds, compounds **5c, 5 g** and **5h** reversed the Aβ-induced cell inhibition, which was consistently with the results in H_2_O_2_-induced model (Figure 3A).

The structure-activity relationship governing this neuroprotection is closely linked to the electrophilic reactivity of the α,β-unsaturated carbonyl motif of the ferulic acid scaffold, which proposed as a electrophilic centre of facilitate key interactions with nucleophilic cysteine residues in Keap1, thereby promoting the activation of the Nrf2-mediated antioxidant defense pathway. Compounds bearing electron-withdrawing groups such as 4-NO_2_ (**5 g**) or 2-Cl (**5h**) exhibited robust neuroprotection (86.52% and 84.54% against H_2_O_2_; 84.15% and 86.12% against Aβ, respectively). Compound 5c, featuring a 2-fluoro substituent, also maintained strong activity (81.10% and 78.35%). Conversely, unsubstituted analogues (**5a**) or those with weaker electron-withdrawing groups showed diminished protection, underscoring the critical role of electron withdrawal. This indicated that increase the electron deficiency of the conjugated system, rendering the β-carbon more electrophilic and thus more favourable for the activation of Nrf2 system.

However, electronic effects alone do not dictate potency. The steric profile of the amino group also plays a decisive role. While dimethylamino or mixed methyl/ethyl substitutions were well tolerated and associated with both high activity and low cytotoxicity, introduction of bulky diethylamino groups (compounds **5k–5o**) led to a marked erosion of neuroprotective efficacy, with most derivatives in this subset failing to exceed 55% viability. This sharp decline suggests that excessive steric bulk may hinder cellular uptake, target accessibility, or optimal binding geometry within the Keap1 binding pocket.

Based on these facts, compounds **5c**, **5 g** and **5h** were selected for the further biological evaluation. Results in Figures 2 and 3(A,B) indicated that compound **5c, 5 g** and **5h** inhibited cell growth inhibition induced by H_2_O_2_ and Aβ in a bell-shaped manner and the best effects were found in 5 μM. Compound **5c, 5 g** and **5h** caused a much more significant elevation in cell viability for all concentrations than FA in Aβ-induced (Figures 2B and 3B).

### Antioxidant activity

The accumulation of Aβ leads to the increase of intracellular ROS level, which could be detected with DCFH. As shown in Figure 3, after treated with 10 μM Aβ, the ROS level were 10.70 times to the control group. While treated with 10 μM Aβ combined with 5 μM compound **5c**, **5g** and **5h** and **FA** respectively, the fluorescence intensities were reduced significantly. Thus, these results indicated that **5c**, **5g** and **5h** and **FA** could reverse the ROS accumulation induced by Aβ.

### Activation of Nrf2 pathway

**Figure 4.**
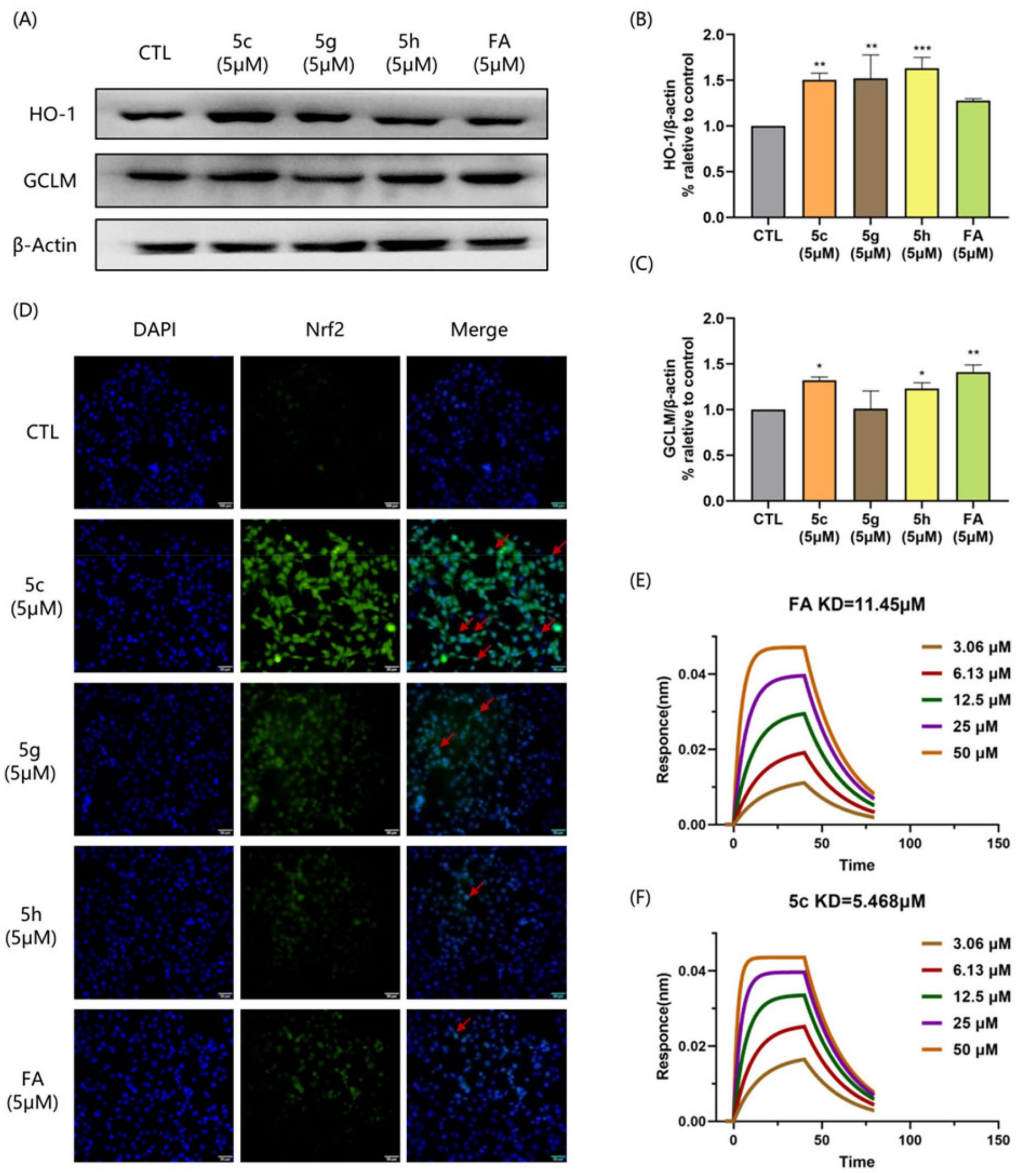
Compound **5c**, **5g**, **5h** and **FA** stimulated Nrf2 pathway. (A) Compound **5c**, **5g**, **5h** and **FA** induced increases in HO-1 and GCLM levels. (B) and (C) Relative protein levels were measured using the control group as the standard. *p < 0.05, **p < 0.01, ***p < 0.001 compared with the control group. (D) Immunofluorescence images showing compound **5c**, **5g** and **5h** and **FA** promoting Nrf2 expression and nuclear translocation (marked with arrows). scale bars, 50 µm; (E) and (F) Bio-layer interferometry assay results of **FA** and **5c** to Keap1 upon different concentrations ranging from 3.06 to 50 nM.

The further study the possible mechanism of these compounds, the Nrf2-ARE pathway activating effect has been taken into consideration since FA has been described as an Nrf2 inducer. As shown in Figure 4(A), all the tested compounds (**5c, 5g, 5h** and **FA**) can significantly induced the expression of HO-1 and GCLM, which are ARE gene known to be activated at the transcriptional level by Nrf2. Compared with FA, compound **5c** and **5h** presented better activity especially on HO-1. Next, considering that the translocation of Nrf2 to the nucleus is the key to the activation of the Nrf2-ARE pathway, we assessed the effect of these compounds (5 μM) on the nuclei translocation of Nrf2 (Figure 4D). After treated with compounds **5c**, **5g**, **5h** and **FA** respectively for 6h, the Nrf2 level was elevated and translocated to nuclear (marked with arrows). Among them, compound **5c** presented much better activity than others.

To elucidate the mechanism of Nrf2 activation, we assessed the direct molecular interaction between compound **5c** and **FA** with Keap1 using biolayer interferometry (BLI). Both **FA** and the **5c** exhibited concentration-dependent binding to Keap1, consistent with a 1:1 binding model (Figure 4E,F). Quantitative analysis revealed that the intact derivative 5c bound with approximately two-fold higher affinity than **FA**, with calculated dissociation constants KD of 5.47 µM and 11.45 µM, respectively. This stronger affinity for **5c** was driven by a faster association rate (Ka = 8.04 × 10³ M^−1^s^−1^) compared to FA (Ka = 3.86 × 10³ M^−1^s^−1^). These results provide direct biophysical evidence that the designed hybrid molecule **5c** engages the Keap1 protein effectively in its intact form, supporting the conclusion that the observed Nrf2 pathway activation is primarily mediated by the parent compound itself.

### In vivo study on C. elegans AD model

In contrast to mammalian models such as mice, which are costly, time consuming, and ethically constrained for early stage screening, the use of transgenic *C. elegans* models offers a distinctive advantage by enabling rapid, medium throughput functional evaluation of neuroprotection and behavioural recovery in a simplified yet intact nervous system, where complex and interrelated pathological cascades, including Aβ aggregation, oxidative stress, and neuronal dysfunction, can be simultaneously engaged and monitored in a live organism. Based on previous study, compounds **5c, 5g,** and **5h** were selected as prioritised candidates for subsequent biological evaluation. Notably, although **5e** exhibited superior BuChE selectivity (SI = 294.14) and potent enzyme inhibition, its relatively weak neuroprotective activity, particularly in the H_2_O_2_-induced oxidative stress model (53.57% viability), limited its potential as a multifunctional agent.

**Figure 5.**
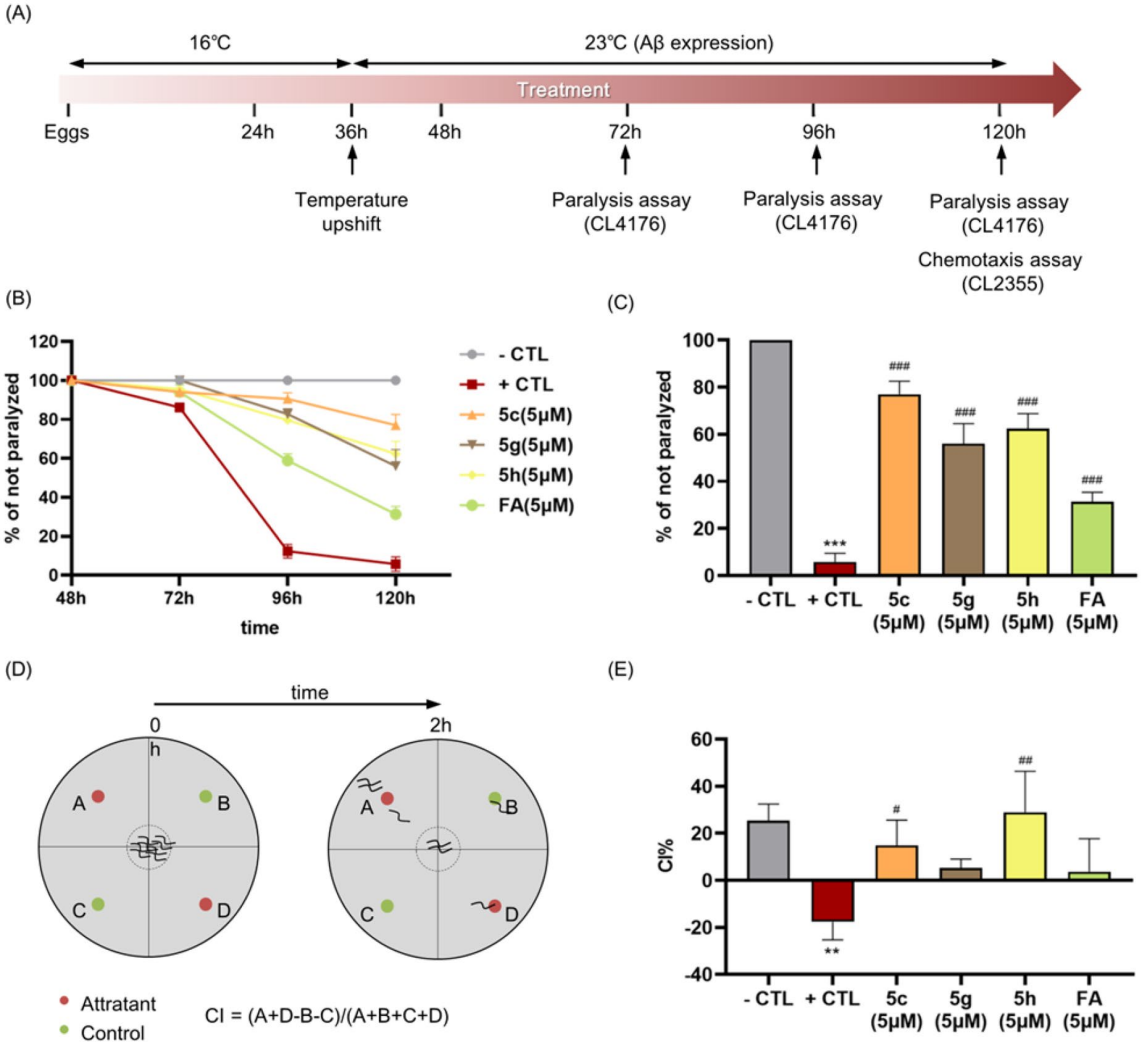
The protection effect of compounds **5c, 5g, 5h** and FA effect against Aβ-induced paralysis in *C. elegans* strain CL4176 and CL2355. (A) Diagram illustrating the paralysis assay and chemotaxis assay showing the time at which the temperature were raised in CL4176 and CL2355 worms (B) Time course of Aβ-induced paralysis in the transgenic CL4176 strain treated by compounds **5c, 5g, 5h** and FA.(C) The paralysis assays were quantified at 120h. (D) Schematic diagram of the chemotaxis assay in CL2355. (E) The Chemotactic Index (CI) in CL2355 treated by compounds **5c, 5g, 5h** and FA. **p < 0.01, ***p < 0.001 vs the negative control group; #*p* < 0.05, ##*p* < 0.01, ###*p* < 0.001 compared with the positive control group.

Compounds **5c, 5g, 5h** and FA were firstly assessed for their protection effect against Aβ-induced paralysis in *C. elegans* strain CL4176 and CL2355. The duration of treatment and behaviour study in CL4176 was shown in Figure 5(A). CL4176 expresses Aβ_42_ in body wall muscles and shows a phenotype of Aβ expression and aggregation in muscles upon a temperature upshift from 15 °C to 23 °C, leading to progressive paralysis. Compared with negative control, the untreated group all paralysed at 120h (84h after temperature upshift). All the tested compounds can significantly delay the Aβ_42_-induced paralysis at 10 μM. At 120h, all the treated group presented a paralysis rate less than 75%. Among them, worms treated by compound **5c, 5g** and **5h** paralysed less than 50%, much better than FA (Figure 5B,C).

Than we investigated the neuroprotective effects of Compounds Compounds **5c, 5g, 5h** and FA on Aβ-induced toxicity in the neurons in *C. elegans* strain CL2355, which utilises Aβ expression from the pan-neuronal synaptobrevin promoter with expression permitted by temperature-sensitive nonsense-mediated RNA degradation. Details of the experiment are illustrated in Figure 5(D). Chemotaxis index (CI) was used as a measure to evaluate attractive (positive value) and repulsive (negative value) responses. After temperature shift, the untreated group showed significant chemosensory deficits and all the tested compounds could reversed this Aβ-induced toxicity. The beat rescue was observed in group treated by compound **5c** and **5h** with CI more than 10%, even comparable with negative control group.

### In silico study

**Figure 6.**
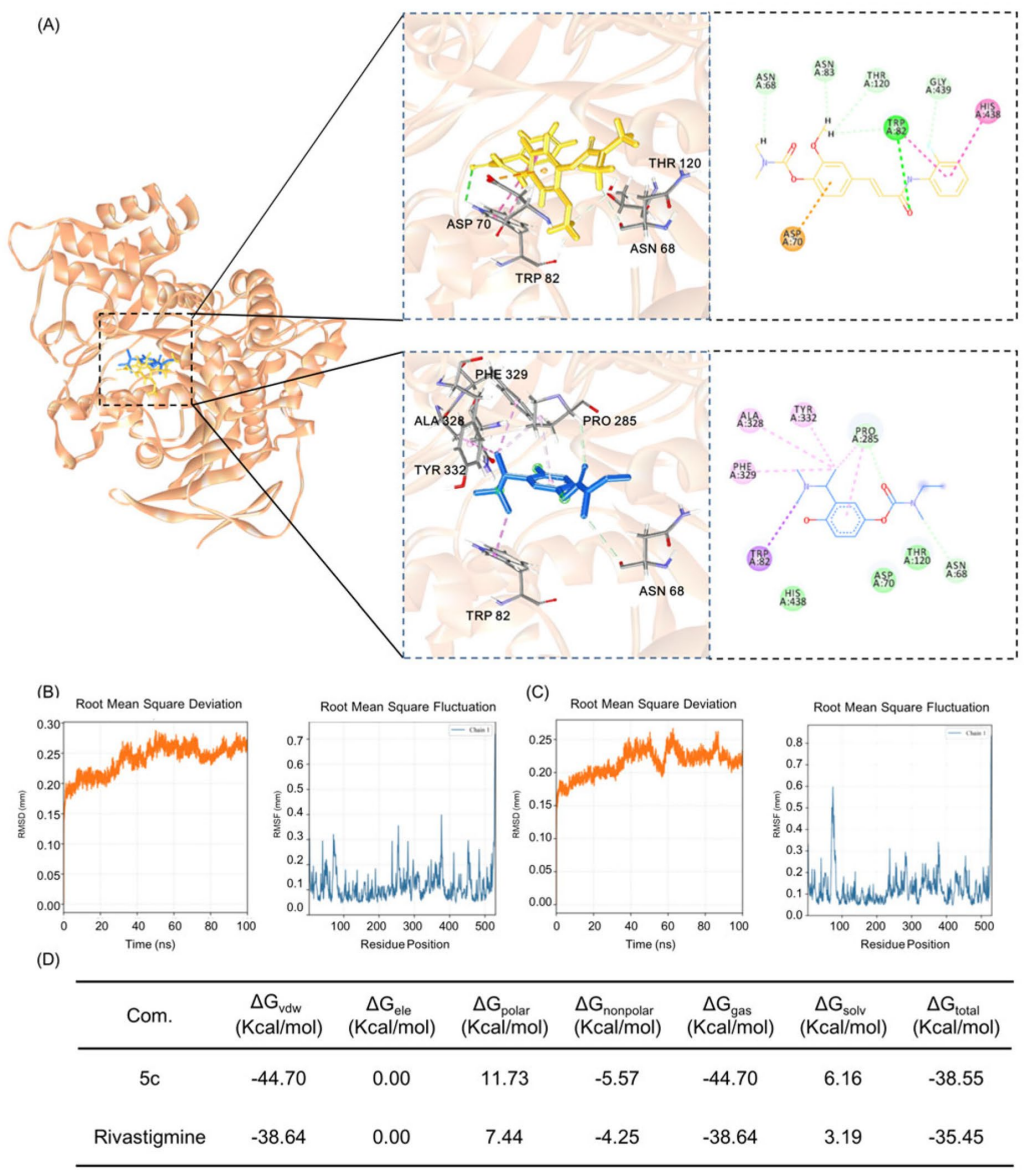
(A) The docking results of Compound 5c (yellow) and rivastigmine (blue) with BuChE (brown, PDB: 6EYF). (B) The RMSD and of RMSF of 5c-BuChE (PDB: 6EYF) complex during 100 ns MDs simulation. (C) The RMSD and of RMSF of rivastigmine-BuChE (PDB: 6EYF) complex during 100 ns MDs simulation. (D) MMPBSA analysis of 5c-BuChE and rivastigmine-BuChE complex.

To rationalise the prospective activities of compound **5c**, molecular docking studies were performed using the Discovery Studio 3.0/CDOCKER protocol. Due to the limited space in the AChE pocket (PDB: 4EY4), no viable docking poses were obtained for **5c**. Figure 6(A) illustrates the docking orientations and interactions of both rivastigmine and compound **5c** with BuChE (PDB: 6EYF). Both ligands favourable positioned within the catalytic active pocket, forming hydrogen bonds with Asn68 through their carbamate substitutions. This observation suggests that **5c** may inhibit BuChE via a resembling mechanism of rivastigmine. Moreover, compared to rivastigmine, **5c** demonstrated enhanced stabilisation through π-π and π-hydrogen interactions with His438, Trp82, and Asp70, in addition to hydrogen bonding with Asn83, Asn68, Thr120, and Gly439.

To gain deeper insights into the conformational dynamics of the complexes formed by compound 5c and the reference molecule rivastigmine with BuChE, we performed 100 ns molecular dynamics (MD) simulations. The root-mean-square deviation (RMSD) analysis indicates that both complexes reached equilibrium during the simulation but exhibited different stability profiles (Figure 6B,C, left panel). The RMSD of the rivastigmine-BuChE complex stabilised at approximately 0.20–0.25 nm during the mid-to-late stages of the simulation, showing minimal overall fluctuation and suggesting a relatively rigid bound conformation. For the 5c-BuChE complex, the RMSD remained primarily within the range of 0.15–0.20 nm after equilibration. Although a slight increase was observed during some periods, the values were consistently low, indicating a stable binding state.

Analysis of the root-mean-square fluctuation (RMSF) of local residues further elucidates the structural basis for the differences in binding stability (Figure 6B,C, right panel). The overall RMSF profile of the 5c–BuChE complex exhibited markedly reduced flexibility, with a maximum peak below 0.35 nm. This indicates that the extended interaction network of **5c** effectively stabilises not only the active site but also restrains flexibility in distal protein regions. In contrast, rivastigmine binding led to pronounced flexibility in several non-active loops, where RMSF peaks approached 0.65 nm, consistent with its more localised interaction pattern near the catalytic centre. Notably, compound **5c** demonstrated a more pronounced local stabilising effect within and around the key catalytic active site of BuChE. In residues corresponding to active site, including Asn68, Asp70, Trp82, Asn83, Thr120, His438 and Gly439, the RMSF values for the 5c-BuChE complex are generally lower than those for the rivastigmine system. This suggests that **5c** more effectively restricts conformational fluctuations in and around the active pocket, enhancing local structural rigidity.

To assess binding energetics, the Molecular Mechanics–Poisson Boltzmann Surface Area approach was employed to rescore each complex using the MMPBSA module (Figure 6D). Binding free energies were decomposed into van der Waals, electrostatic, polar solvation, and solvent-accessible surface area contributions across three independent simulation replicates. The results revealed that Compound **5c** exhibited a more favourable total binding free energy of −38.55 kcal/mol relative to −35.45 kcal/mol for rivastigmine, indicating a better binding affinity. This improvement is primarily driven by stronger van der Waals interactions and more favourable nonpolar solvation energy, despite a higher polar solvation penalty. These results suggest that 5c possesses superior binding characteristics and warrants further investigation as a potential lead compound.

**Figure 7.**
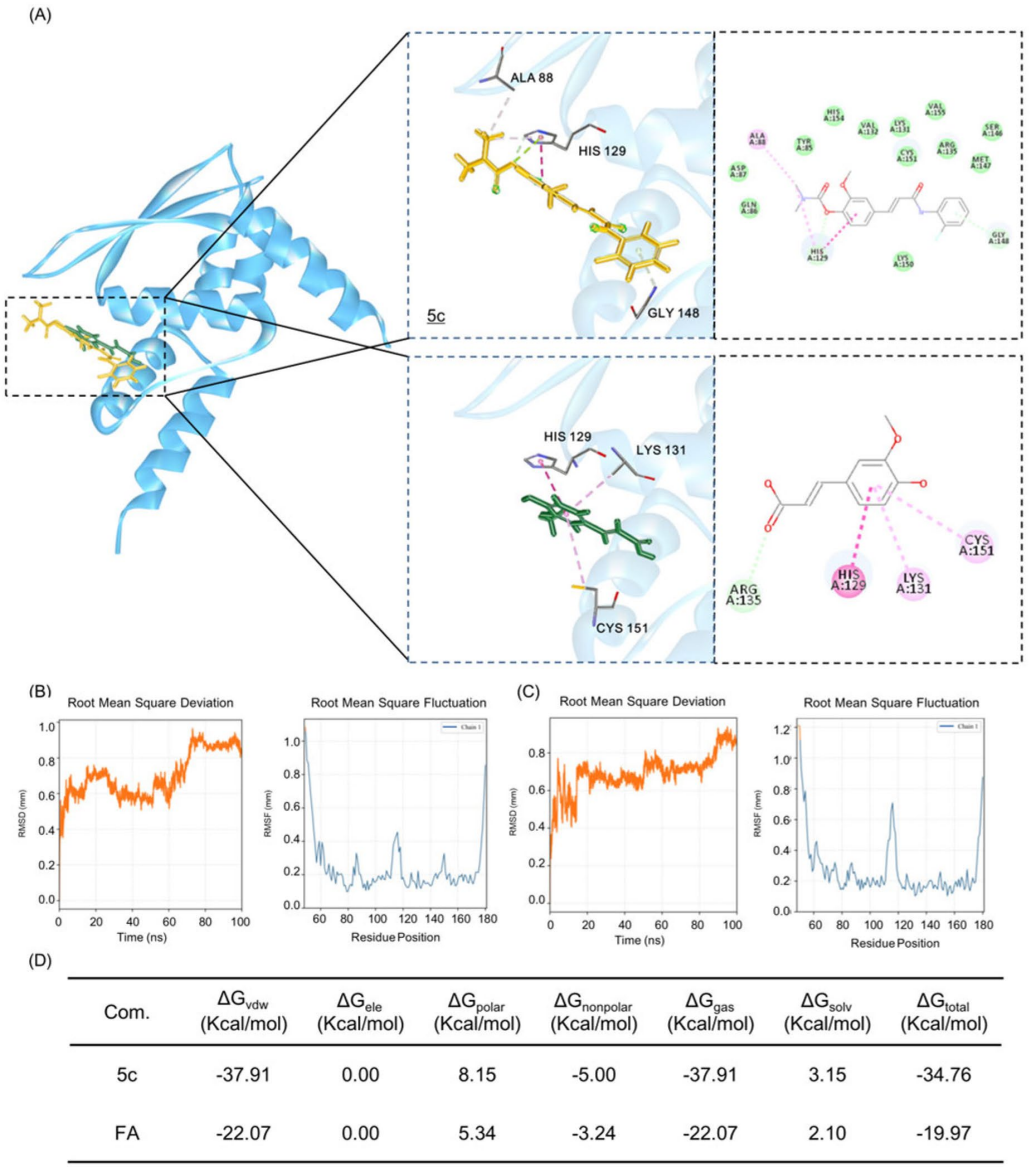
(A) The docking results of Compound 5c (yellow) and FA (green) with Keap1 (blue, PDB: 4CXT). The docking results of Compound 5c (yellow) and FA (green) with Keap1 (cyan, PDB: 4CXT). (B) The RMSD and of RMSF of 5c-Keap1 (PDB: 6EYF) complex during 100 ns MDs simulation. (C) The RMSD and of RMSF of FA-Keap1 (PDB: 6EYF) complex during 100 ns MDs simulation. (D) MMPBSA analysis of 5c-Keap1 and Rivastigmine-Keap1 complex.

Classical Nrf2 agonists exert their effects by covalently interaction with Cys151, Cys273, and Cys288 residues on Keap1, thereby disrupting the Keap1-Cul3 complex conformation^33^. The binding site is a large positively charged area named 3-box domain. To elucidate the mechanism of Nrf2 pathway activation, we conducted docking studies with Keap1 (PDB: 4CXT), as depicted in Figure 7(A). Intriguingly, both **5c** and FA exhibited interactions with Cys151. Notably, 5c adopted a more extended conformation that optimally occupied the 3-box cavity and formedπ-π and π-hydrogen interactions with Ala88 and His129, in addition to hydrogen bonding with Gly148.

The MD results also demonstrate that the 5c-Keap1 complex exhibits superior structural stability compared to the FA, consistent with its more favourable binding mode predicted by molecular docking (Figure 7B,C, left panel). Analysis of the overall conformation revealed that the RMSD of the 5c-Keap1 complex remained at a lower and more stable level throughout the simulation, indicating a relatively rigid binding conformation without significant conformational drift. In contrast, the FA-Keap1 complex showed higher RMSD values with more pronounced fluctuations, suggesting greater overall protein flexibility in its bound state.

Further investigation into local residue mobility, via RMSF, elucidated the structural basis for this stability difference (Figure 7B,C, right panel). The binding of 5c significantly reduced the conformational flexibility of key functional regions in Keap1 especially within the 3-box domain, approximately residues 88–151, which is critical for Nrf2 binding. The structural fluctuations were notably reduced in regions adjacent to key residues, including Ala88 involved in a π-π interaction, His129 participating in a π-hydrogen bond, as well as the hydrophobic pocket encompassing Gly148 which is hydrogen-bonded and the covalently targeted Cys151. This multipoint anchoring effect aligns well with the docking results, wherein the extended conformation of 5c optimally occupies the 3-box cavity, forming an extensive and stable interaction network. Conversely, the RMSF profile of the FA-Keap1 complex showed higher peaks of fluctuation near key residues, reflecting its weaker stabilisation of the local structure and incomplete restriction of binding pocket flexibility.

The further MMPBSA calculations indicated that compound 5c displayed a total binding free energy of −34.76 kcal/mol, markedly lower than that of FA (−19.97 kcal/mol), indicating substantially stronger binding affinity (Figure 7D). Compound 5c exhibited a van der Waals energy of −37.91 kcal/mol, substantially stronger than that of FA (−22.07 kcal/mol). This suggests that 5c achieves better shape complementarity with the binding cavity or forms more extensive hydrophobic contacts and dispersion interactions. These energetic features underscore the superior binding profile of compound 5c and warrant its further investigation in this target system.

The physicochemical profiles and the ADME properties of compound **5c** and FA were predicted by SwissADME software (http://www.swissadme.ch/)^34^. The results suggested good adequate oral bioavailability and drug-like proprieties (Table 3). All the compounds have suitable molecular weight (MW) (<500 Da), polar surface area (TPSA) (<140 A), number of hydrogen bond acceptors (HBA) (<10), number of hydrogen bond doners (HBD) (<5), and number of rotatable bonds (RB) (<10). The structural modifications leaded to higher LogP and lower LogS in compound **5c**, which revealed that compound **5c** can cross the BBB, while FA cannot.

**Table 3.** The physicochemical profiles and the ADME properties predicted by SwissADME software.

Com.	MW	RB	HBA	HBD	TPSA	LogP	LogS	GI	BBB	Lipinski
**5c**	358.36	8	5	1	67.87	3.56	−3.78	High	Yes	Yes
FA	194.18	3	4	2	66.76	1.62	−2.11	High	No	Yes

## Discussion and conclusion

The multifactorial pathogenesis of AD urged for therapeutic strategies that address interconnected pathological cascades, including cholinergic dysfunction, oxidative stress, and Aβ toxicity. Emerging evidence highlights that BuChE activity increases as AD progresses, making it a relevant target for symptomatic treatment, while selective inhibition may circumvent the peripheral side effects associated with AChE inhibition. Concurrently, activating the Nrf2 antioxidant pathway offers a strategy to enhance endogenous defense against oxidative damage. In this study, we rationally integrated selective BuChE inhibition and Nrf2 activation into a single molecular scaffold, thereby testing the feasibility of a synergistic approach to combat AD progression.

Among these, compounds 5c and 5e were identified as the most potent BuChE inhibitors, exhibiting over 150-fold selectivity for BuChE over AChE. Remarkably, the introduction of aniline moieties at the carboxyl terminus not only optimised calculated lipophilicity, suggesting improved potential for BBB penetration but also enhanced selectivity to acyl-binding pockets of BuChE relative to AChE, providing a structural rationale for their enhanced inhibitory potency and selectivity.

The dual functionality of these compounds was further validated through their potent neuroprotection in HT22 cells. The reverse of cell viability in both H_2_O_2_^−^ and Aβ-induced toxicity models (84.54%–86.52% and 78.80%–82.21%, respectively) demonstrated their capacity to mitigate oxidative damage and Aβ pathology. Mechanistically, the observed upregulation of HO-1 and GCLM via Nrf2 nuclear translocation confirmed that the compounds function as indirect antioxidants by activating this critical cellular defense mechanism.

The in vivo potential of lead compounds **5c, 5g** and **5h** was further evaluated in two well-established *C. elegans* AD models. At 10 μM, these derivatives reduced Aβ-induced paralysis over 50% in CL4176, much better than FA. Concurrently, they rescued chemotaxis dysfunction with CI comparable with negative control in CL2355. Remarkably, their potency surpassed ferulic acid by 5–10 fold, underscoring the success of aniline-driven structural optimisation in enhancing BBB permeability and dual-target engagement. These results set a new benchmark for multifunctional AD therapeutics, bridging biochemical efficacy with functional restoration at non-toxic doses.

In summary, this study successfully designed and characterised a novel series of ferulic acid carbamate derivatives that integrate selective BuChE inhibition with Nrf2 antioxidant pathway activation within a single molecular scaffold. Strategic structural optimisation, particularly through aniline substitution at the carboxyl terminus, yielded compounds that effectively balance dual-target engagement, potent neuroprotection, and favourable drug-like properties, including optimised lipophilicity for potential blood–brain barrier penetration. A key structural insight underlying the selectivity of these compounds lies in the differential architecture of the cholinesterase active sites. The acyl-binding pocket of BuChE is inherently larger and more accommodating than that of AChE. The introduction of sterically hindered aniline groups at the carboxyl terminus exploits this topological difference, fitting preferentially into the more spacious BuChE pocket while being excluded from the narrower AChE site. This design rationale, which combines a rivastigmine-inspired carbamate motif with the ferulic acid pharmacophore for Keap1 interaction, distinguishes our approach from previous hybrids that focused primarily on reversible inhibition and general antioxidant capacity. The therapeutic potential of this multi-target strategy was validated in vivo using two well-established *C. elegans* AD models. The lead compounds significantly alleviated both Aβ-induced progressive paralysis and chemosensory cognitive deficits, providing direct evidence of their disease-modifying potential beyond conventional *in vitro* assays. Collectively,.

In spite of these findings establish a robust proof-of-concept for synergistically integrating cholinergic regulation with cellular defense mechanisms, several limitations of the present study should be acknowledged. While BLI result supported the binding of the intact molecule to Keap1, the in vivo stability and metabolic profile of this carbamate series warrant further investigation. Comprehensive pharmacokinetic studies are required to elucidate the metabolic fate and active species responsible for the observed effects. Additionally, although the *C. elegans* model offers utility for initial in vivo screening, its physiological divergence from mammalian systems represents an inherent limitation. Extrapolation of these findings to higher organisms therefore necessitates validation in appropriate mammalian models to substantiate the translational relevance.

Looking forward, this study lays a solid foundation for developing disease-modifying AD therapeutics. Future efforts should prioritise detailed pharmacokinetic and pharmacodynamic profiling in mammalian models, investigation of long-term efficacy and safety in chronic dosing studies, and exploration of the compounds’ potential influences on other AD hallmarks, such as tau pathology.

## Experimental

### Chemistry

Melting points of compounds were measured on a RY-1G melting point apparatus and were uncorrected. Nuclear magnetic resonance (^1^H NMR) spectra were recorded on a Bruker AV-300 (300 MHz) spectrometer as deuterochloroform (CDCl_3_) solutions using tetramethylsilane (TMS) as an internal standard (δ = 0) unless noted otherwise. Electron impact mass spectral (EI-MS) data were obtained on a SHIMADZU GCMS-QP2010 system. All chemicals were purchased from commercial sources and were used without further purification unless otherwise noted. The solvents (such as MeOH, EtOAc, EtOH, CH_2_Cl_2_ and others) were C.P. grade purchased from Nanjing Chemical Co., Ltd. and used without further purification. Column chromatography (CC) was carried out on silica gel (200–300 mesh, Qingdao Ocean Chemical Company, China). Thin-layer chromatography (TLC) analyses were carried out on silica gel GF254 (Qingdao Ocean Chemical Company, China) glass plates (2.5 cm × 10 cm with 250 μm layer). Concentration and evaporation of the solvent after reaction or extraction was carried out on a rotary evaporator operated at reduced pressure.

#### Synthesis of methyl (E)-3–(4-hydroxy-3-methoxyphenyl) acrylate (2)

FA (3 g, 15.5 mmol) was disolved in 30 ml MeOH with H_2_SO_4_ added slowly. The mixture stirred at 70 °C for 3 h. 10% NaOH was added to adjust PH to 7 in ice bath. The reaction mixture was extracted three times with ethyl acetate. It was dried with sodium sulphate and the solvent was removed in vacuum provide crude product. Recrystallized by acetone to provide white solid 2.6 g, yield 81.38%.^1^H-NMR (400 MHz, CDCl_3_) *δ* 7.64(1H, d, *J* = 16.0 Hz, H-7), 7.08(1H, d, *J* = 8.2 Hz, H-5), 7.04(1H, s, H-3), 6.93 (1H, d, *J* = 8.0 Hz, H-6), 6.31(1H, d, *J* = 16.0 Hz, H-8), 6.07 (1H, brs, OH), 3.93 (3H, s, OCH_3_), 3.82 (3H, s, OCH_3_); ESI-MS *m/z*: 208.21 [M + H]^+^。

#### General procedure for the preparation of (E)-3–(4-(carbamoyloxy-3-methoxyphenyl) acrylic acid derivatives(4a–4c)

To the solution of 2 (1 g. 4.8 mmol) and K_2_CO_3_ (662 mg, 4.8 mmol) in 10 ml acetone, 1.1eq corresponding carbamyl chloride was added. The mixture was stirred at 60 °C for 7h. After cooled to room temperature, 30 ml water was added and the reaction mixture was extracted three times with ethyl acetate. It was dried with sodium sulphate and the solvent was removed in vacuum to give crude product (3a–3c).

To the solution of corresponding methyl acrylate product (3a–3c) in 15 ml THF and 5 ml H_2_O, 3eq LiOH was added. The mixture was stirred at r.t. for 24h. The reaction mixture was added into 30 ml H_2_O after evaporate the majority of solvent. 10% HCl was added to adjust PH to 4 and the reaction mixture was extracted three times with ethyl acetate. It was dried with sodium sulphate and the solvent was removed in vacuum provide crude product. Recrystallized by methanol to provide white solid.

##### (E)-3–(4-((dimethylcarbamoyl)oxy)-3-methoxyphenyl)acrylic acid(4a)

Yield 75.50% of pure product. mp 201 °C–202 °C; ^1^H-NMR (400 MHz, CDCl_3_) *δ* 12.39(1H, s, COOH), 7.60(1H, d, *J* = 16.0 Hz, H-7), 7.46(1H, s, H-3), 7.25 (1H, d, *J* = 8.0 Hz, H-5), 7.11 (1H, d, *J* = 8.0 Hz, H-6), 6.58(1H, d, *J* = 16.0 Hz, H-8), 3.83(3H, s, OCH_3_), 3.05(3H, s, –CH_3_), 2.91(3H, s, –CH_3_); ESI-MS *m/z*: 266.1 [M + H]^+^.

##### (E)-3–(4-((ethyl(methyl)carbamoyl)oxy)-3-methoxyphenyl)acrylic acid(4b)

Yield 78.34% of pure product. mp 126 °C–128 °C; ^1^H-NMR (400 MHz, CDCl_3_) *δ* 12.41(1H, s, COOH), 7.59(1H, d, *J* = 16.0 Hz, H-7), 7.47(1H, s, H-3), 7.25 (1H, d, *J* = 8.0 Hz, H-5), 7.11 (1H, d, *J* = 8.0 Hz, H-6), 6.59(1H, d, *J* = 16.0 Hz, H-8), 3.83(3H, s, OCH_3_), 3.01(5H, d, –CH_2_, –CH_3_), 1.19(3H, t, –CH_3_); ESI-MS *m/z*: 280.1 [M + H]^+^.

##### (E)-3–(4-((diethylcarbamoyl)oxy)-3-methoxyphenyl)acrylic acid(4c)

Yield 76.28% of pure product. mp 126 °C–127 °C; ^1^H-NMR (400 MHz, CDCl_3_) *δ* 7.72(1H, d, *J* = 16.0 Hz, H-7), 7.13, 7.10(3H, d, *J* = 12 Hz, H-3,5,6), 6.36(1H, d, *J* = 16.0 Hz, H-8), 3.87(3H, s, OCH_3_), 3.40(4H, dd, –CH_2_), 1.24(6H, tt, –CH_3_); ESI-MS *m/z*: 294.1 [M + H]^+^.

#### General procedure for the preparation of (E)-2-methoxy-4–(3-oxo-3-(phenylamino)-1-propenyl)phenyllcarbamate (5a–5o)

In the solution of 4a–4c (2 mmol), HATU (616 mg, 2.4 mmol) and DIEPA (0.33 ml, 4 mmol) in DCM, 2 mmol corresponding aniline was added after stirred for 15 min. The mixture was stirred for 2h. Upon completion, the reaction mixture was poured into 30 ml water and extracted with ethyl acetate. The combined organic layers were dried and the solvent was evaporated in vacuum to provide yellow oil. The compound was purified by column chromatography (PE/acetone, 105:30).

##### (E)-2-methoxy-4–(3-oxo-3-(phenylamino)-1-propenyl)phenyldimethyl carbamate (5a)

Yield 78.34% of pure product; Purity: 96.26%; mp 178 °C–180 °C;^1^H-NMR (400 MHz, CDCl_3_) *δ* 8.34(1H, s, H-9), 7.65 (2H, d, *J* = 8.0 Hz, H-11,15), 7.47(1H, d, *J* = 16 Hz, H-7), 7.32(2H, t, H- 12,14), 7.09(1H, t, H-13), 7.04(1H, d, *J* = 8.0 Hz, H-4), 6.96(1H, d, *J* = 8.0 Hz, H-3), 6.89 (1H, s, H-6), 6.00(1H, d, *J* = 16 Hz, H-8), 3.66(3H, s, OCH_3_), 3.19(3H, s, –CH_3_), 3.10(3H, s, –CH_3_); ^13^C NMR (101 MHz, CDCl_3_) δ 176.26, 172.57, 171.96, 154.48, 152.10, 148.52, 147.25, 146.94, 146.49, 142.73, 140.36, 132.52, 126.71, 123.86, 123.69, 123.45, 121.71, 118.95, 117.27, 114.90, 114.62, 111.47, 110.14, 109.61, 79.72, 77.44, 77.12, 76.81, 57.06, 56.10, 56.05, 43.57, 36.95, 36.71, 0.08. HRMS (ESI) m/z: calcd for C_19_H_21_N_2_O_4_ [M + H]^+^ 341.1501, Found 341.1506.

##### (E)-4–(3-((4-chlorophenyl)amino)-3-oxo-1-propenyl)-2-methoxyphenyldimethyl carbamate (5b)

Yield 60.74% of pure product; Purity: 95.64%; mp164-166 °C; ^1^H-NMR (400 MHz, CDCl_3_) *δ* 8.52(1H,s, H-9), 7.62(2H, d, *J* = 8.0 Hz, H-11,15), 7.42(1H, d, *J* = 16 Hz, H-7), 7.28(2H, d, H-12, 14), 7.04(1H, d, *J* = 8.0 Hz, H-4), 6.93(1H, d, *J* = 8.0 Hz, H-3), 6.84(1H, s, H-6), 5.82(1H, d, *J* = 16 Hz, H-8), 3.62(3H, s, OCH_3_), 3.20(3H, s, –CH_3_), 3.11(3H, s, –CH_3_); ^13^C NMR (101 MHz, CDCl_3_) δ 164.19, 155.36, 151.51, 141.27, 140.40, 138.97, 133.82, 128.91, 123.88, 123.05, 121.39, 120.10, 119.81, 112.82, 77.44, 77.12, 76.81, 55.73, 53.53, 37.01, 36.82, 0.09. HRMS (ESI) m/z: calcd for C_19_H_20_N_2_O_4_Cl [M + H]^+^ 375.1112, Found 375.1122. ESI-MS *m/z*: 375.0 [M + H]^+^。

##### (E)-2-methoxy-4–(3-((4-nitrophenyl)amino)-3-oxo-1-propenyl) phenyldimethyl carbamate(5c)

Yield 44.84% of pure product; Purity: 95.82%; mp 168 °C–169 °C; ^1^H-NMR (400 MHz, CDCl_3_) *δ* 8.74(1H, dd, *J* = 8 Hz, H-12), 8.45(1H, dd, *J* = 8.0 Hz, H-14), 8.22(1H, d, *J* = 8.0 Hz, H-11), 8.04(1H, d, *J* = 16 Hz, H-7), 7.83(2H, d, *J* = 8.0 Hz, H-15), 7.45(1H, dd, *J* = 8.0 Hz, H-11), 7.24(1H, dd, *J* = 8.0 Hz, H-4)7.19(1H, s, H-6), 7.18(2H, d, *J* = 8.0 Hz, H-3), 6.72(1H, d, *J* = 16 Hz, H-8), 3.91(3H, s, OCH_3_), 3.15(3H, s, –CH_3_), 3.04(3H, s, –CH_3_); ^13^C NMR (101 MHz, CDCl_3_) δ 164.08, 155.49, 151.42, 141.17, 140.39, 137.78, 133.84, 128.85, 128.57, 122.94, 121.15, 120.93, 119.85, 113.20, 77.44, 77.12, 76.80, 55.65, 53.53, 37.01, 36.85, 0.08. HRMS (ESI) m/z: calcd for C_19_H_20_N_3_O_6_ [M + H]^+^ 386.1352, Found 386.1362.

##### (*E*)-4–(3-((2-bromophenyl)amino)-3-oxo-1-propenyl)-2-methoxyphenyl dimethylcarbamate (5d)

Yield 37.50% of pure product; Purity: 95.34%; mp 175 °C–176 °C; ^1^H-NMR (400 MHz, CDCl_3_) *δ* 8.75(1H, dd, *J* = 8 Hz, H-11), 8.45(1H, dd, *J* = 8.0 Hz, H-14), 8.04(1H, d, *J* = 16 Hz, H-7), 7.45(1H, dd, *J* = 8.0 Hz, H-12), 7.25(1H, dd, *J* = 8.0 Hz, H-4)7.19(1H, s, H-6), 7.18(2H, d, *J* = 8.0 Hz, H-3),6.72(1H, d, *J* = 16 Hz, H-8), 3.91(3H, s, OCH_3_), 3.14(3H, s, –CH_3_), 3.03(3H, s, –CH_3_); ^13^C NMR (101 MHz, CDCl_3_) δ 169.92, 162.85, 154.19, 152.33, 151.86, 150.97, 143.89, 140.74, 135.15, 131.65, 129.66, 124.14, 123.82, 122.43, 121.60, 120.98, 117.21, 111.86, 111.41, 110.66, 77.45, 77.13, 76.81, 56.19, 56.09, 36.95, 36.73, 29.79, 0.08. HRMS (ESI) m/z: calcd for C_19_H_20_N_2_O_4_Br [M + H]^+^ 419.0606, Found 419.0609.

##### (E)-4–(3-((2-fluorophenyl)amino)-3-oxo-1-propenyl)-2-methoxyphenyl dimethylcarbamate (5e)

Yield 74.17% of pure product; Purity: 99.01%; mp 174 °C–175 °C;^1^H-NMR (400 MHz, CDCl_3_) *δ* 8.45(1H, t, H-9), 7.97(1H, s, H-11), 7.59(1H, d, *J* = 16 Hz, H-7), 7.16(1H, d, *J* = 8.0 Hz, H-12), 7.12(1H, d, *J* = 8.0 Hz, H-14), 7.07(1H, d, *J* = 8.0 Hz, H-13), 7.05(2H, s, H-3,6), 6.99(1H, s, H-4), 6.40(1H, d, *J* = 16 Hz, H-8), 3.80(3H, s, OCH_3_), 3.15(3H, s, –CH_3_), 3.04(3H, s, –CH_3_); 13 C NMR (101 MHz, CDCL3) δ 162.85, 154.17, 152.34, 151.87, 150.97, 143.90, 142.63, 140.75, 135.15, 132.37, 131.64, 129.65, 128.56, 124.15, 123.81, 122.44, 121.17, 120.97, 111.86, 111.59, 110.65, 77.45, 77.13, 76.81, 56.20, 56.16, 36.95, 36.73, 0.09. HRMS (ESI) m/z: calcd for C_19_H_20_N_2_O_4_F [M + H]^+^ 359.1407, Found 359.1413.

##### (E)-2-methoxy-4–(3-oxo-3-(phenylamino)-1-propenyl)phenylethyl (methyl)carbamate (5f)

Yield 64.87% of pure product; Purity: 98.59%; ^1^H-NMR (400 MHz, CDCl_3_), *δ* 8.48(1H, s, H-9), 7.65 (2H, d, *J* = 8.0 Hz, H-11,15), 7.44(1H, d, *J* = 16 Hz, H-7), 7.32(2H, t, H- 12,14), 7.08(1H, d, *J* = 8.0 Hz, H-13), 7.04(1H, d, *J* = 8.0 Hz, H-4), 6.94(1H, d, *J* = 8.0 Hz, H-3), 6.85(1H, s, H-6), 5.93(1H, d, *J* = 16 Hz, H-8), 3.62(3H, s, OCH_3_), 3.55(2H, q, –CH_2_), 3.12(3H, d, –CH_3_), 1.29(3H, t, –CH_3_); ^13^C NMR (101 MHz, CDCl_3_) δ 164.23, 154.96, 153.89, 151.78, 151.47, 141.93, 141.75, 133.27, 126.96, 126.86, 124.68, 124.64, 124.31, 124.24, 123.50, 122.24, 120.98, 120.70, 114.98, 114.79, 111.99, 77.45, 77.13, 76.81, 55.96, 36.94, 36.75, 0.09. HRMS (ESI) m/z: calcd for C_20_H_23_N_2_O_4_ [M + H]^+^ 355.1658, Found 355.1661.

##### (E)-4–(3-((2-fluorophenyl)amino)-3-oxo-1-propenyl) −2-methoxyphenylethyl methyl)carbamate (5g)

Yield 55.62% of pure product; Purity: 97.48%; ^1^H-NMR (400 MHz, CDCl_3_) *δ* 8.43(1H, s, H-9), 7.92(1H, d, H-11), 7.60(1H, d, *J* = 16 Hz, H-7), 7.16(1H, d, *J* = 8.0 Hz, H-4), 7.12(1H, d, *J* = 8.0 Hz, H-12), 7.07(3H, d, *J* = 8.0 Hz, H-3,6,14), 7.00(1H, d, H-13), 6.40(1H, d, *J* = 16 Hz, H-8), 3.80(3H, s, OCH_3_), 3.55(2H, q, –CH_2_), 3.12(3H, d, –CH_3_), 1.25(3H, t, –CH_3_); ^13^C NMR (101 MHz, CDCl_3_) δ 167.49, 153.79, 152.07, 144.57, 142.61, 132.61, 123.80, 121.33, 117.57, 111.31, 108.56, 77.45, 77.13, 76.81, 56.02, 51.80, 42.41, 42.29, 42.16, 14.09, 13.44, 11.29, 0.08. ESI-MS *m/z*: 373.1[M-H]^+^.

##### (E)-4–(3-((2-bromophenyl)amino)-3-oxo-1-propenyl)-2-methoxyphenylethyl(methyl)carbamate (5h)

Yield 77.44% of pure product; Purity: 95.01%; mp 141 °C–142 °C; 1H-NMR (400 MHz, CDCl_3_) *δ* 8.75(1H, dd, *J* = 8 Hz, H-11), 8.45(1H, dd, *J* = 8.0 Hz, H-14), 8.05(1H, d, *J* = 16 Hz, H-7), 7.45(1H, dd, *J* = 8.0 Hz, H-12), 7.26(1H, s, H-6,), 7.19(2H, s, H-3),6.72(1H, d, *J* = 16 Hz, H-8), 3.91(3H, s, OCH_3_), 3.46(2H, q, –CH_2_), 3.15(3H, d, –CH_3_), 1.25(3H, t, –CH_3_); ^13^C NMR (101 MHz, CDCl_3_) δ 171.85, 154.24, 154.04, 152.13, 146.44, 142.85, 142.73, 132.50, 123.90, 123.83, 121.71, 117.26, 111.48, 111.41, 77.45, 77.13, 76.81, 56.09, 56.04, 44.41, 44.33, 34.50, 34.05, 13.06, 12.48. ESI-MS *m/z*: 434.1 [M + H]^+^.

##### (E)-4–(3-((2-chlorophenyl)amino)-3-oxo-1-propenyl)-2-methoxyphenylethyl(methyl) carbamate (5i)

Yield 64.29% of pure product; Purity: 97.96%; 1H-NMR (400 MHz, CDCl_3_) *δ* 8.75(1H, dd, *J* = 8 Hz, H-11), 8.45(1H, dd, *J* = 8.0 Hz, H-14), 7.72(1H, d, *J* = 16 Hz, H-7), 7.45(1H, dd, *J* = 8.0 Hz, H-12), 7.26(1H, s, H-4,), 7.19(1H, s, H-6),6.53(1H, d, *J* = 16 Hz, H-8), 3.89(3H, s, OCH_3_), 3.45(2H, dd, –CH_2_), 3.05(3H, d, –CH_3_), 1.24(3H, t, –CH_3_); ^13^C NMR (101 MHz, CDCl_3_) δ 171.86, 153.86, 152.13, 146.53, 142.89, 132.35, 123.88, 121.73, 117.15, 111.45, 77.44, 77.12, 76.81, 56.03, 42.45, 42.19, 14.08, 13.43. ESI-MS *m/z*: 389.1 [M + H]^+^.

##### (E)-2-methoxy-4–(3-((4-nitrophenyl)amino)-3-oxo-1-propenyl)phenylethyl(methyl)carbamate (5j)

Yield 57.11% of pure product; Purity: 96.50%; mp 77 °C–79 °C; ^1^H-NMR (400 MHz, CDCl_3_) *δ* 8.90(1H, s, H-9), 8.22(2H, d, *J* = 9.2 Hz, H-12,14), 8.04(1H, d, *J* = 16 Hz, H-7), 7.84(2H, d, *J* = 9.2 Hz, H-11,15), 7.19(1H, d, *J* = 8.0 Hz, H-4), 7.09(1H, dd, *J* = 8.0 Hz, H-6), 6.97(1H, dd, *J* = 8.0 Hz, H-3), 6.73(1H, d, *J* = 16 Hz, H-8), 3.90(3H, s, OCH_3_), 3.45(2H, q, –CH_2_), 3.12(3H, t, –CH_3_), 1.28(3H, t, –CH_3_); ^13^C NMR (101 MHz, CDCl_3_) δ 169.78, 162.86, 153.91, 153.73, 152.36, 151.86, 151.01, 145.96, 144.05, 143.94, 140.74, 135.15, 131.60, 129.65, 128.55, 124.19, 124.10, 122.45, 120.98, 111.81, 110.60, 77.46, 77.14, 76.82, 56.15, 44.44, 44.35, 34.50, 34.08, 13.08, 12.48, 0.08. HRMS (ESI) m/z: calcd for C_20_H_22_N_3_O_6_ [M + H]^+^ 400.1509, Found 400.1516.

##### (E)-2-methoxy-4–(3-oxo-3-(phenylamino)-1-propenyl)phenyldiethylcarbamate (5k)

Yield 88.88% of pure product; Purity: 95.27%; mp 140 °C–142 °C; ^1^H-NMR (400 MHz, CDCl_3_) *δ* 8.41(1H, s, H-9), 7.65 (2H, d, *J* = 8.0 Hz, H-11,15), 7.45(1H, d, *J* = 16 Hz, H-7), 7.32(2H, t, H- 12,14), 7.08(1H, d, *J* = 8.0 Hz, H-13), 7.05(1H, d, *J* = 8.0 Hz, H-4), 6.97(1H, d, *J* = 8.0 Hz, H-3), 6.86(1H, s, H-6), 5.92(1H, d, *J* = 16 Hz, H-8), 3.63(3H, s, OCH_3_), 3.50(4H, q, 2× –CH_2_), 1.30(6H, tt, –CH_3_); ^13^C NMR (101 MHz, CDCl_3_) δ 164.54, 162.85, 155.35, 151.87, 151.49, 150.98, 145.18, 143.07, 141.59, 140.73, 135.16, 133.55, 129.68, 124.96, 124.16, 123.10, 122.45, 121.00, 120.54, 120.07, 119.15, 113.36, 111.84, 110.62, 77.44, 77.12, 76.81, 56.15, 55.67, 44.66, 44.36, 34.64, 34.52, 34.12, 13.09, 12.81, 12.48, 0.08. HRMS (ESI) m/z: calcd for C_21_H_25_N_2_O_4_ [M + H]^+^ 369.1814, Found 369.1817.

##### (E)-4–(3-((2-chlorophenyl)amino)-3-oxo-1-propenyl)-2-methoxy phenyldiethylcarbamate (5l)

Yield 51.79% of pure product; Purity: 98.03%; mp 124 °C–125 °C;^1^H-NMR (400 MHz, CDCl_3_) *δ* 8.74(1H, dd, *J* = 8.0 Hz, H-11), 8.45(1H, dd, *J* = 8.0 Hz, H-14), 8.05(1H, d, *J* = 16.0 Hz, H-7), 7.45(1H, dd, *J* = 8.0 Hz, H-12), 7.23(1H, d, *J =* 12.0 Hz, H-4), 7.19(1H, s, H-6), 6.72(1H, d, *J* = 16 Hz, H-8), 3.91(3H, s, OCH_3_), 3.43(4H, q, 2×–CH_2_), 1.24(6H, tt, –CH_3_); ^13^C NMR (101 MHz, CDCl_3_) δ 164.17, 154.91, 151.53, 141.26, 140.23, 139.06, 133.78, 129.01, 128.82, 123.78, 123.06, 121.36, 120.03, 119.88, 112.91, 77.46, 77.14, 76.82, 56.99, 55.92, 55.63, 42.32, 42.08, 14.03, 13.61. HRMS (ESI) m/z: calcd for C_21_H_24_N_2_O_4_Cl [M + H]^+^ 403.1425, Found 403.1427. ESI-MS *m/z*: 403.1 [M + H]^+^.

##### (E)-2-methoxy-4–(3-(4-nitrophenyl)amino)-3-oxo-1-propenyl)phenyl diethylcarbamate (5m)

Yield 42.93% of pure product; Purity: 95.17%; mp 125 °C–126 °C;^1^H-NMR (400 MHz, CDCl_3_) *δ* 8.74(1H, dd, *J* = 8 Hz, H-11), 8.45(1H, dd, *J* = 8.0 Hz, H-14), 8.05(1H, d, *J* = 16 Hz, H-7), 7.46(1H, dd, *J* = 8.0 Hz, H-12), 7.22(1H, d, *J* = 12 Hz, H-3), 7.19(1H, s, H-6)6.72(1H, d, *J* = 16 Hz, H-8), 3.91(3H, s, OCH_3_), 3.43(4H, q, 2×–CH_2_), 1.24(6H, tt, –CH_3_); ^13^C NMR (101 MHz, CDCl_3_) δ 162.87, 153.58, 152.36, 151.86, 151.03, 144.06, 140.74, 135.15, 131.48, 129.65, 124.97, 124.15, 122.46, 120.97, 119.16, 111.83, 110.52, 77.46, 77.14, 76.82, 56.13, 42.50, 42.25, 29.14, 26.99, 22.71, 14.10, 13.43, 11.53, 0.08. HRMS (ESI) m/z: calcd for C_21_H_24_N_3_O_6_ [M + H]^+^ 414.1665, Found 414.1667.

##### (E)-4–(3-((2-bromophenyl)amino)-3-oxo-1-propenyl)-2-methoxy phenyldiethylcarbamate (5n)

Yield 58.17% of pure product; Purity: 95.97%; mp 100 °C–102 °C;^1^H-NMR (400 MHz, CDCl_3_) *δ* 8.75(1H, dd, *J* = 8 Hz, H-11), 8.45(1H, dd, *J* = 8.0 Hz, H-14), 8.04(1H, d, *J* = 16 Hz, H-7), 7.45(1H, dd, *J* = 8.0 Hz, H-12), 7.25(1H, d, H-6,), 7.20(2H, d, H-4,13), 7.09(1H, d, H-3), 6.72(1H, d, *J* = 16 Hz, H-8), 3.90(3H, s, OCH_3_), 3.43(4H, q, 2×–CH_2_), 1.25(6H, tt, –CH_3_); ^13^C NMR (101 MHz, CDCl_3_) δ 170.20, 162.87, 153.87, 153.58, 152.36, 152.09, 151.86, 151.04, 145.96, 144.06, 142.68, 142.46, 140.74, 135.97, 135.15, 132.71, 132.46, 132.37, 131.47, 129.66, 128.54, 125.34, 124.16, 123.82, 122.46, 121.60, 121.16, 120.98, 120.40, 117.31, 111.83, 111.60, 111.38, 110.52, 77.46, 77.14, 76.82, 56.13, 56.09, 56.02, 42.50, 42.24, 38.84, 14.10, 13.43, 0.08. HRMS (ESI) m/z: calcd for C_21_H_24_N_2_O_4_Br [M + H]^+^ 447.0919, Found 447.0925.

##### (E)-4–(3-((2-fluorophenyl)amino)-3-oxo-1-propenyl)-2-methoxy phenyldiethylcarbamate (5o)

Yield 50.36% of pure product; Purity: 98.06%; ^1^H-NMR (400 MHz, CDCl_3_) *δ* 8.43(1H, s, H-9), 7.93(1H, s, H-11), 7.60(1H, d, *J* = 16 Hz, H-7), 7.15(1H, d, *J* = 8.0 Hz, H-6), 7.09(1H, d, *J* = 8.0 Hz, H-12), 7.07(2H, d, *J* = 8.0 Hz, H-3,14), 7.01(1H, s, H-13), 6.40(1H, d, *J* = 16 Hz, H-8), 3.81(3H, s, OCH_3_), 3.45(4H, q, 2×–CH_2_), 1.23(6H, tt, –CH_3_); ^13^C NMR (101 MHz, CDCl_3_) δ 162.87, 153.56, 152.36, 151.86, 151.04, 144.08, 140.75, 135.15, 131.47, 129.64, 124.16, 122.46, 120.97, 111.83, 110.52, 77.46, 77.14, 76.82, 56.13, 42.50, 42.24, 14.10, 13.43. ESI-MS *m/z*: 387.1 [M + H]^+^.

### Cholinesterase activity

The target compounds were tested for their inhibitory potency against cholinesterases using Ellman’s protocol. modified for 96-well microplates. All the reagents were purchased from Sigma–Aldrich (Steinheim, Germany). The stock solutions of the target compounds were prepared in DMSO and diluted with water to yield the desired final concentrations.

The enzymes (AChE, E.C.3.1.1.7, from electric eel and BuChE, E.C.3.1.1.8, from equine serum) were prepared as 50 U/mL aqueous stock solutions and diluted before use to a final concentration of 0.1 U/mL. Then 20 μL of prepared enzyme solutions (AChE or BuChE) each was added to the reaction mixture in the wells, containing 20 μL of the target compound (in case of blank samples, water or water/DMSO mixture), 120 μL of PBS and 20 μL of 5,5′ -dithiobis-(2-nitrobenzoic acid) DTNB (5 mM). All those reagents were preincubated for 5 min at 25 °C for the reactions with the animal enzymes (eeAChE or eqBuChe). The enzymatic reaction was initiated by the addition of 20 μL of acetylthiocholine iodide ATC substrate (3.75 mM) or butyrylthiocholine iodide BTC (3.75 mM) solutions (depending on the enzyme used). After 30 min of incubation, changes in absorbance were measured at 412 nm. For compounds showing > 50% inhibition of enzyme (eeAChE or eqBuChE) activity at 50 μM, IC_50_ values were determined measuring absorbance at seven different inhibitor concentrations.

### Cell culture and CCK-8 assay

HT22 Cells was purchased from Procell Life Science & Technology Co., Ltd. (Wuhan, China). Dulbecco’s modified Eagle’s medium (DMEM) media, foetal bovine serum (FBS), and penicillin-streptomycin double antibiotics were purchased from Procell Life Science & Technology Co., Ltd. (Wuhan, China). HT22 were cultured in DMED medium (containing 10% (v/v) FBS, 100 U/mL Penicillin and 100 mg/mL Streptomycin) in a 5% CO_2_-humidified atmosphere at 37 °C. Cells were trypsinized and seeded at a density of 1 × 10^5^/mL into a 96-well plate (100 ml/well) and incubated at 37 °C, 5% CO_2_ atmosphere for 24 h. After this time they were treated with 100 ml/well medium containing test compounds which had been pre-prepared to provide the concentration range of 100 μM, 10 μM, 1 μM and 0.1 μM, and re-incubated for a further 48 h. Control wells were added the equivalent volume of medium containing 1% (v/v) DMSO. Cell viability was assessed using the CCK-8 kit (C008-3, 7sea biotech, China) following the manufacturer’s instructions. CCK-8 solution was added 20 μL for each wall and continued to incubate in darkness at 37 °C for 1 h. The optical density values were read at 450 nm.

### ROS detection

ROS generation was determined using a ROS Assay Kit (Beyotime, China). After incubation with or without Aβ for 24 h, the cells were stained with 10 μM DCFH-DA at 37 °C for 20 min and then imaged using a fluorescence microscope.

### Western blot analysis

Proteins were prepared using a total protein extraction kit (Invent, China) containing protease and phosphatase inhibitors (Roche, Switzerland). Protein concentrations were determined using a BCA protein assay (Thermo Scientifc, USA). Equal amounts of proteins (20 μg) were separated using 15% SDS-PAGE and transferred to polyvinylidene fluoride membranes (Millipore, USA). The membranes were blocked with 10% non-fat dry milk for 1 h, incubated with primary antibodies overnight at 4 °C, washed with TBST buffer and incubated at room temperature for 1 h with an HRP-conjugated anti-rabbit antibody (1:10,000, Zhuangzhi Biology, China). The primary antibodies were an anti-GCLM antibody (1:2000, Proteintech 66808) and an anti-HO-1 antibody (1:2000, Abcam ab189491). β-Actin was used as an internal control and detected using an anti-β-actin antibody (1:20,000, Proteintech 66009). The blots were detected using Immobilon Western Chemiluminescent HRP Substrate (Millipore, USA). The proteins were visualised using the ChemiDoc^™^ Touch Imaging System (Bio-Rad, USA), and the proteins were quantifed by Image Lab 5.1 software.

### Immunofuorescence

For cultured cells, immunofuorescence was performed as previously described (35). The following primary antibodies were used for immunofuorescence: Nrf2 (1:200, Proteintech 16396). After thorough washes in PBST, sections or cells were incubated with 1:1000 dilution of FITC-conjugated secondary antibodies (1:200, Zhuangzhi Biology, China) appropriate for the species of the primary antibodies, followed by DAPI counterstains. The images acquired using Leica-LMD7000 confocal laser-scanning confocal microscopes and analysed by Image-J software.

### Biolayer interferometry (BLI) assay

The binding affinities of tested compounds for Keap1 were determined using Octet^®^ R8 system (Sartorius AG, Göttingen, Germany). Octet NTA Biosensors captured with purified His-tag Keap1 (50 μg/ml) or without proteins were used as ligand sensors or reference sensors, respectively. For binding kinetic measurements, the association and dissociation of tested compounds (6.125–100 μM) in assay buffer (PBS, 0.02% Tween 20, 2% DMSO) were monitored at 25 °C with 1,000 rpm stirring for 90 s. For accurate baseline, wells containing assay buffer only were used as reference wells. All data were analysed by Octet BLI analysis software version 12.2 (Sartorius AG, Göttingen, Germany). The binding graphs were obtained using a double reference subtraction protocol and equilibrium dissociation constant (KD) values were calculated from the ratio of the dissociation rate constant (kdis) to the association rate constant (ka)

### In vivo study on C. elegans AD model

The CL4176 and CL2355 *C. elegans* strains was obtained from Suny Biotech, with detailed information available on wormbase.org. These strains were cultivated at 20 °C on nematode growth medium (NGM) agar plates, supplemented with live Escherichia coli OP50 bacteria to serve as a food source.

#### Paralysis assay

The paralysis assay was conducted according to the schematic shown in Figure 5(A). In brief, age-synchronised CL4176 transgenic strains were cultured at 16 °C on NGM plates (60 mm × 10 mm) seeded with either an OP50 suspension in 100 mM ammonium bicarbonate buffer (serving as the negative control) or the same vehicle containing compound 5c, 5g, 5h, or FA. Each plate contained approximately 20 eggs. Aβ transgene expression in body wall muscle was induced by shifting the temperature from 16 °C to 23 °C at 36 h post-egg laying. Paralysis was assessed at 24-h intervals beginning 12 h after temperature upshift, and continued until roughly 80% of worms in the negative control group became paralysed. Worms were classified as paralysed if they failed to move or exhibited only head movement when stimulated with a platinum loop. The time-dependent progression of paralysis was recorded and graphed.

#### Chemotaxis assay

The chemotaxis assay was performed as illustrated in Figure 5(A,D). Briefly, synchronised transgenic CL2355 strains were maintained at 16 °C on NGM plates (90 mm × 15 mm culture plates). They were spotted with an OP50-containing vehicle (100 mM ammonium bicarbonate buffer) and with or without 5c, 5g, 5h and FA. For the assay, eggs were maintained at 16 °C for 36 h (∼20–60 eggs/plate), and then temperature was upshifted to 23 °C–25 °C to induce expression of neuronal Aβ and the eggs were incubated for another 36 h. Worms were then collected, washed with M9 buffer thrice, and assayed on 90 mm plates containing 1.9% agar, 1 mM CaCl_2_, 1 mM MgSO_4_, and 25 mM phosphate saline buffer. Worms were placed at the centre, and 2 μL of 0.1% benzaldehyde in 100% ethanol and 1 μL of sodium azide were added at positions A and D as attractant odorants (Figure 4A). 100% ethanol (2 μL) and sodium azide (1 μL) were added at positions B and C as control odorants. Assay plates were incubated at 23 °C–25 °C for 2 h and CI was calculated using the formula: CI = [(number of worms at the attractant position- numbers of worms at the control position)/total number of worms scored)]

### Molecular modelling

The molecular modelling was performed with Discovery Studio.3.0/CDOCK protocol (Accelrys Software Inc.). The crystal structures of BuchE (PDB: 6EYF) and Keap1 (PDB: 4CXT) were downloaded from Protein Data Bank. Compound **5c**, FA and rivastigmine were drowned and optimised using Hyperchem v7.0. The protein and ligand were optimised and charged with CHARMm force field to perform docking. Up to 10 conformations were retained, and binding modes presented graphically are representative of the highest-scored conformations.

### Molecular dynamics (MD)

To elucidate the binding dynamics, MD simulations were performed using AMBER. Atomic charges of the ligands were derived from Gaussian 09 calculations. Each complex was solvated in a TIP3P water box and neutralised with Na^+^ and Cl^−^. Energy minimisation (5000 steps, steepest descent) was followed by 500 ps NVT and 500 ps NPT equilibration at 310 K. A 100 ns production run was then conducted under NPT/NVT ensembles with a 2 fs time step. Post-simulation analyses including RMSD, RMSF, and MM-PBSA binding free energy were employed to assess system stability and interaction strength.

### Statistical analysis

Statistical analysis of the data gathered in this study was performed using GraphPad Prism software. The data are expressed as the mean ± SEM. Differences between two groups were determined using unpaired Student’s t-test. Differences among more than two groups were assessed by one-way ANOVA, and *post hoc* comparisons were performed by Bonferroni post-test. *p* < 0.05 was regarded as significant.

## Data Availability

The authors confirm that the data supporting the findings of this study are available within the article [and/or] its supplementary materials.
